# Generation of a rod-specific NRL reporter line in human pluripotent stem cells

**DOI:** 10.1038/s41598-018-20813-3

**Published:** 2018-02-05

**Authors:** M. Joseph Phillips, Elizabeth E. Capowski, Andrew Petersen, Alex D. Jansen, Katherine Barlow, Kimberly L. Edwards, David M. Gamm

**Affiliations:** 1 Waisman Center, University of Wisconsin-Madison, Madison, WI USA; 2 McPherson Eye Research Institute, University of Wisconsin-Madison, Madison, WI USA; 3 Department of Ophthalmology and Visual Sciences, University of Wisconsin-Madison, Madison, WI USA

## Abstract

Reporter lines generated in human pluripotent stem cells can be highly useful for the analysis of specific cell types and lineages in live cultures. We created the first human rod reporter line using CRISPR/Cas9 genome editing to replace one allele of the Neural Retina Leucine zipper (*NRL*) gene with an *eGFP* transgene in the WA09 human embryonic stem cell (hESC) line. After confirming successful targeting, three-dimensional optic vesicle structures were produced to examine reporter specificity and to track rod differentiation in culture. The *NRL*^+/eGFP^ hESC line robustly and exclusively labeled the entirety of rods throughout differentiation, eventually revealing highly mature structural features. This line provides a valuable tool for studying human rod development and disease and testing therapeutic strategies for retinitis pigmentosa.

## Introduction

Human pluripotent stem cell (hPSC) reporter lines permit identification of cell lineages and subtypes with visualization of morphology in live cultures. They are also useful for immunocytochemical (ICC) analysis of fixed cultures when cell type-specific and/or cytoplasmic antibodies are not available. While multiple mouse reporter models exist that endogenously label neural retina (NR) cell types^1–5^, few such reporter lines have been generated in hPSCs^6–9^. The particular importance of generating gene reporters in hPSCs is evident from the lack of alternative model systems for studying developing human cells and tissues longitudinally.

From a photoreceptor (PR) standpoint, all hPSC reporter lines generated thus far utilized the cone rod homeobox (*CRX*) gene to drive transgene expression^8,9^. These *CRX-*based hPSC reporters robustly mark all PRs from early to late differentiation. However, they cannot distinguish rods from cones^8,9^, nor are they inherently PR-specific since *CRX* is known to be expressed in other human retinal cell types^10^. In an effort to address these limitations and delineate PR subtypes in hPSC cultures, we developed a rod-specific reporter line by commandeering the endogenous Neural Retina Leucine zipper (*NRL*) promoter. NRL plays a key role in determining rod fate by regulating numerous rod-specific genes^1,11–17^, and in mammals it is expressed in rods from specification through to maturity. Fluorescent reporter genes driven by the *Nrl* promoter have proven robust and specific in labeling rods in mice^1,18^, and have facilitated numerous studies of murine rod development, gene profiling, and transplantation^1,19–23^. To pursue similar studies, an equivalently robust tool created from hPSCs is needed.

Herein, we describe the generation of a targeted *NRL* reporter line in WA09 human embryonic stem cells (*NRL*^+/eGFP^ hESCs) using CRISPR/Cas9 technology. Using a modified version of our previously described optic vesicle (OV) differentiation protocol, we demonstrate production of a high percentage of rods from the *NRL*^+/eGFP^ hESC line that are capable of self-assembling within an organized outer nuclear layer and assuming a mature rod structure replete with developing outer segments, as has been shown previously in unmodified hPSC lines^24–28^. The availability of a *NRL* hPSC reporter line that faithfully and innocuously labels human rods throughout differentiation should prove useful for numerous applications of stem cell technology, particularly those related to the study and treatment of retinitis pigmentosa.

## Results

### WA09 *NRL*^+/eGFP^ hESC line generation

**Figure 1:**
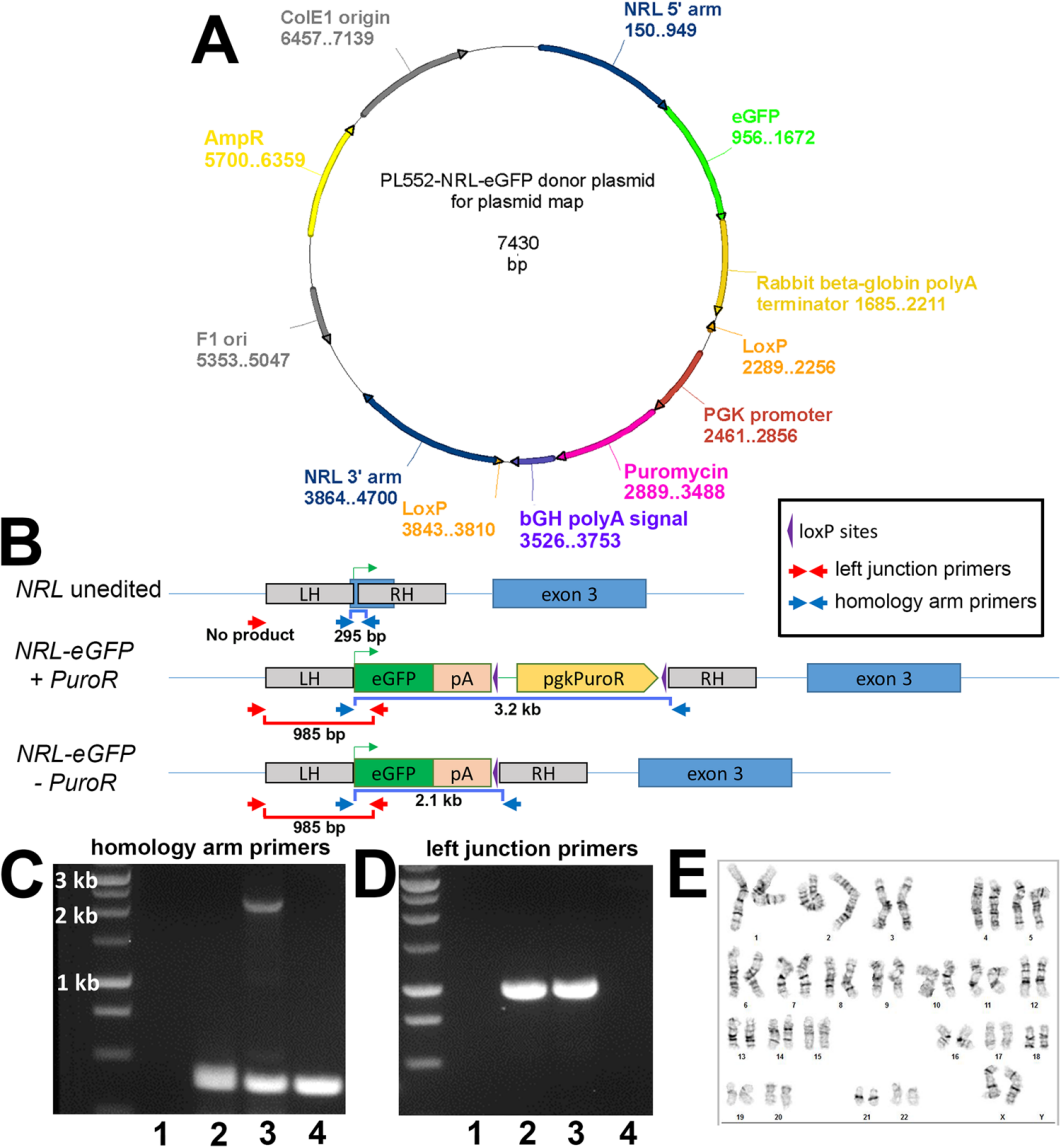
*NRL*^+/eGFP^ hESC line production. (**A**) Donor plasmid map. An *eGFP* coding sequence was fused to the *NRL* start codon, followed by the rabbit beta-globin polyA terminator and a loxP-flanked puromycin resistance (*PuroR*) selection cassette. *NRL* homology arms flanked the genomic sequence on either side of the *NRL* start. (**B**) Schematic of an unedited (wildtype) *NRL* allele (top), an *NRL* allele with the *eGFP* transgene and *PuroR* selection cassette inserted (*NRL*-eGFP+ PuroR reporter; middle), and an *NRL* allele with the eGFP transgene following CRE recombinase-mediated removal of the PuroR selection cassette (*NRL*-eGFP− PuroR; bottom). Primers that amplify DNA located between the left and right homology arms of *NRL* (“homology arm primers”; blue arrows) or DNA spanning the junction between the left homology arm and the GFP transgene (“left junction primers”; red arrows) are shown. (**C**,**D**) Images of agarose gels of genomic PCR products obtained using the homology arm primers (**C**: blue primer set) or the left junction primers (**D**; red primer set). Numbered lanes in panels C and D gels correspond to the same control or clone. Lane 1 = no template control; lane 2 = clone showing the presence of both an unedited *NRL* allele (295 bp fragment in panel C) and an *NRL* allele harboring a targeted insertion of *eGFP* with a *PuroR* cassette (986 bp fragment in panel D and the absence of a 2.1 kb fragment in panel C); lane 3: clone showing the presence of both an unedited *NRL* allele (295 bp fragment in panel C) and an *NRL* allele harboring a targeted insertion of *eGFP* following successful excision of the *PuroR* cassette (986 bp fragment in panel D and 2.1 kb fragment in panel C); lane 4: clone showing the presence of unedited *NRL* alleles only (295 bp fragment in panel C and no amplified product in panel D). (Note that under the PCR conditions used, the 3.2 kb fragment predicted for the *NRL*-*eGFP*+ *PuroR* is not to be amplified. Gels in (**C**,**D**) were cropped for space. The full-length gel is available in Supplementary Information). (**E**) G-banding analysis demonstrating maintenance of a normal karyotype in the *NRL*^+/eGFP^ line.

To generate a rod specific reporter line, we targeted replacement of a single *NRL* allele of the WA09 hESC line with an *eGFP* reporter gene using CRISPR/Cas9-mediated gene editing. A detailed description is provided in Materials and Methods and is summarized here. A donor plasmid was constructed by fusing the *eGFP* coding sequence to the *NRL* start codon followed by the rabbit beta-globin polyA terminator and a loxP-flanked puromycin resistance (*PuroR*) selection cassette (Fig. 1A). Following electroporation of the donor plasmid, a Cas9^D10A^ nickase plasmid, and plasmids containing *NRL* targeting sgRNAs, clones that incorporated the donor sequence were identified by puromycin resistance. Surviving colonies were screened by PCR and one clone was selected for further analysis and removal of the *PuroR* cassette to optimize eGFP expression. Genotyping was performed with primers that distinguished the unedited *NRL* allele from successfully targeted alleles with and without the *PuroR* cassette (Fig. 1B). The selected clone demonstrated targeted incorporation of the *eGFP* reporter transgene at a single *NRL* locus prior to and after successful excision of the *PuroR* cassette (lanes 2 and 3, respectively, in Fig. 1C and D). The *NRL*^+/eGFP^ clone was expanded and fully sequenced to rule out unintended genetic modifications. Thereafter, to ensure that no off-target mutations were introduced, the ten highest-likelihood sites predicted by the crispr.mit.edu algorithms for each sgRNA were amplified and sequenced from the original clone and no off-target indels were detected (see Supplemental Information). Finally, the gene-edited *NRL*^+/eGFP^ line was karyotyped to confirm that the multi-step clonal selection process did not result in chromosomal abnormalities (Fig. 1E).

### Production and isolation of OVs from *NRL*^+/eGFP^ hESCs

**Figure 2:**
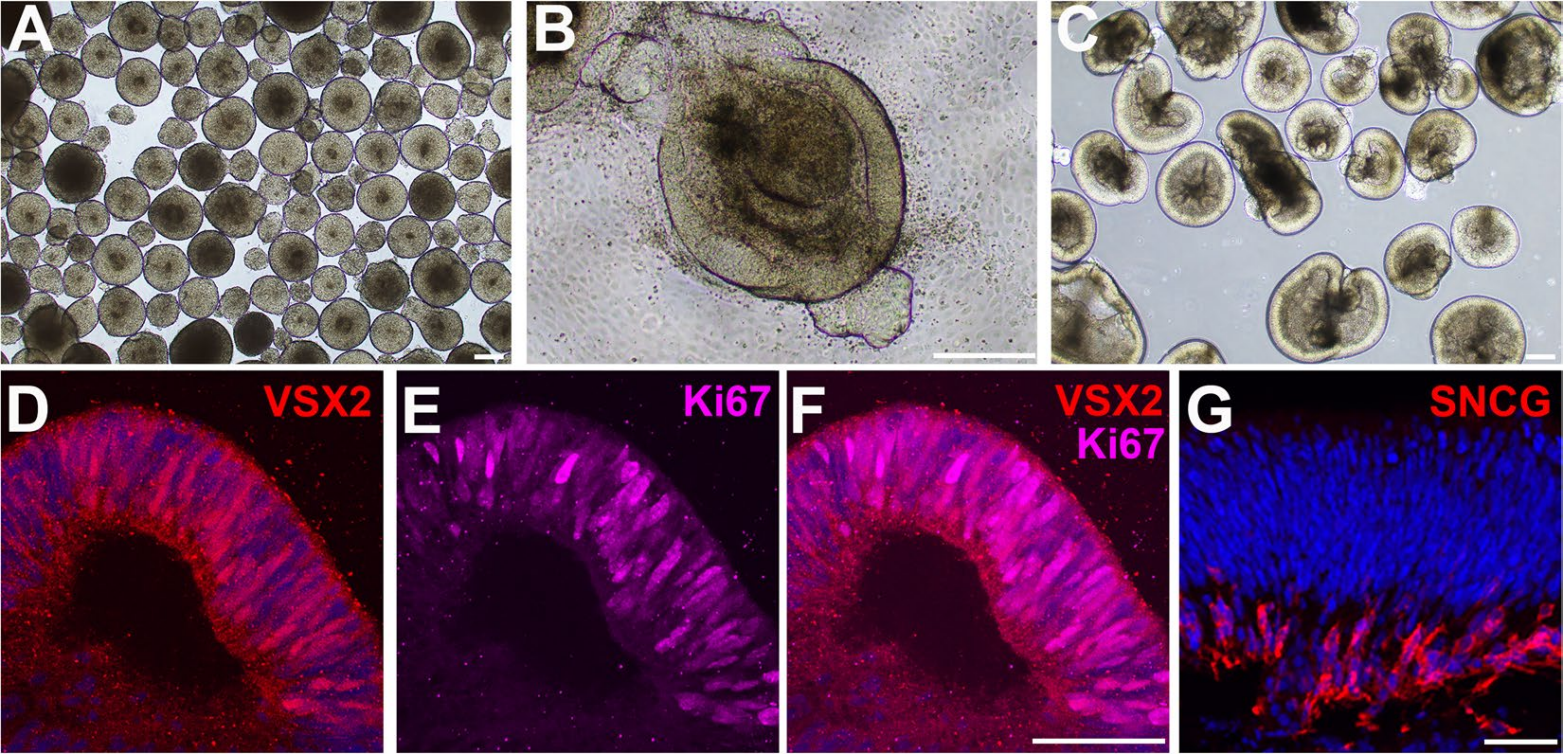
Generation of optic vesicles (OVs) from *NRL*^+/eGFP^ hESCs. (**A**) Free-floating embryoid bodies (EBs) are shown at day (d) 6. (**B**) EBs are plated, whereupon they form OV-like structures (d26 shown). (**C**) After dissection, OVs are maintained long-term in suspension cultures (d30 shown). (**D–G**) At d30, *NRL*^+/eGFP^ OVs consist mainly of an outer layer of VSX2+ (**D**) and Ki67+ (**E**) proliferative retinal progenitor cells (merged image in (**F**), and an inner layer of SNCG+ retinal ganglion cells (**G**). Scale bars in A–C = 100 µm; F, G = 50 µm.

WA09 *NRL*^+/eGFP^ hESCs were lifted on day (d) 0 to form free-floating embryoid bodies (EBs) (Fig. 2A). EBs were subsequently plated on d7 and maintained in retinal differentiation medium (RDM; see Materials and Methods) until d20–30 when early OV colonies were clearly identifiable by light microscopy (Fig. 2B). Following manual dissection, the isolated OVs were maintained in suspension (Fig. 2C) in 3D-RDM for the duration of their time in culture (see Materials and Methods for details of the culture protocol). At d30, OVs consisted mainly of an outer layer of proliferative VSX2 + neural retina progenitor cells (NRPCs) (Fig. 2D–F) and an inner layer of SNCG+ retinal ganglion cells (Fig. 2G).

### Monitoring rod production in live *NRL*^+/eGFP^ OVs

**Figure 3:**
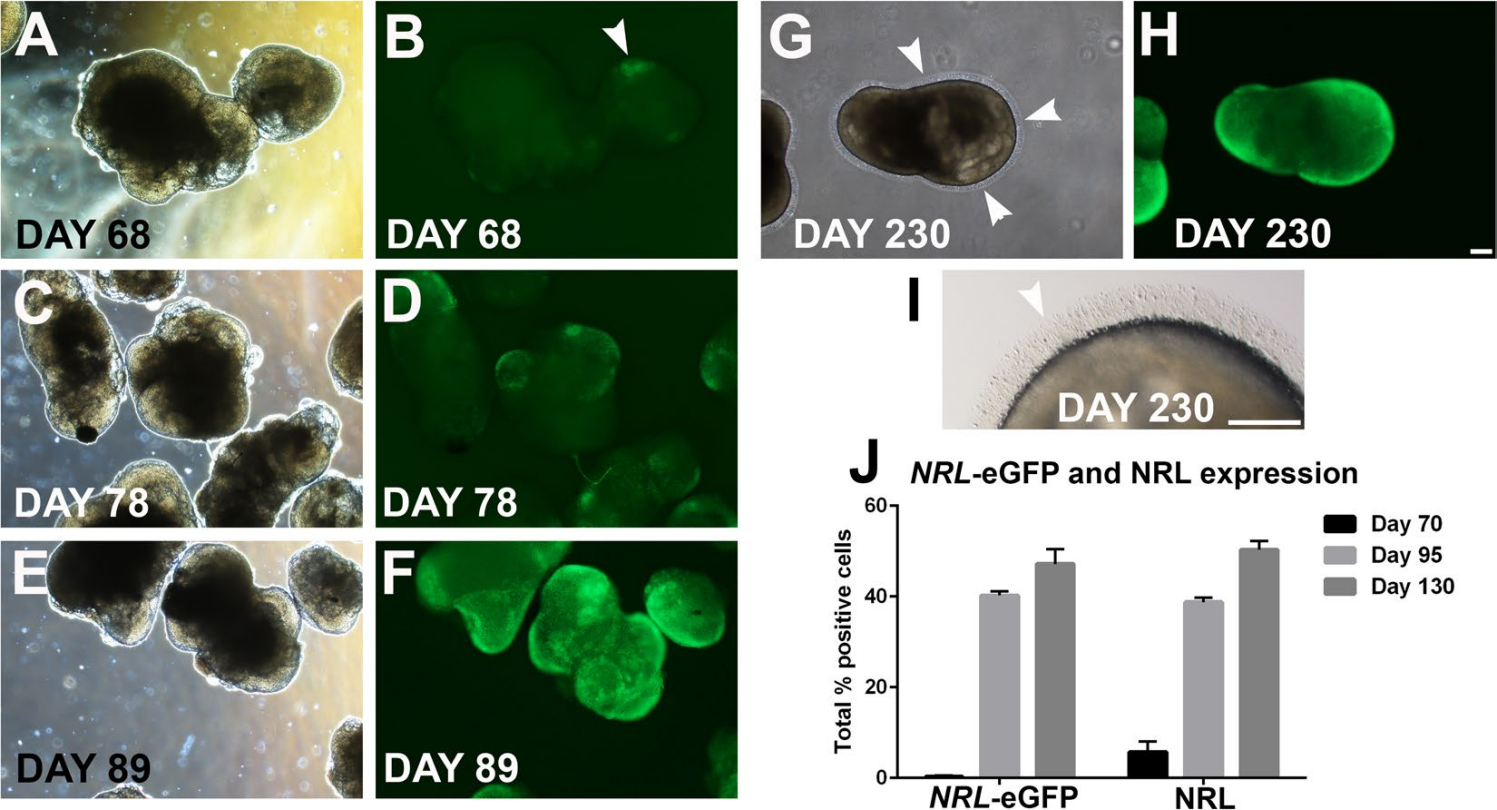
Observing rod production in live *NRL*^+/eGFP^ OVs. Phase and epifluorescence images of live *NRL*^+/eGFP^ OVs were obtained over time in live cultures. (**A**,**B**) Small patches of *NRL*-eGFP were detected beginning at d68 (arrowhead in **B**). (**C–F**) *NRL*-eGFP fluorescence increased from d78 (**C**,**D**) to d89 (**E**,**F**), by which time all OVs demonstrated robust *NRL*-eGFP fluorescence. (**G**,**H**) *NRL*-eGFP fluorescence is maintained to at least d230, the last time point examined. (**G,I**) Hair-like outer segment projections are formed along the outside of OVs (arrowheads). (**J**) To quantify the number of *NRL*-eGFP+ and NRL+ cells, dissociated OVs were plated and immunolabeled with an anti-NRL antibody. The number of *NRL*-eGFP+ and NRL+ cells increased dramatically and concomitantly from d70 to d95. At d130, roughly half of all cells within OVs were immunopositive for *NRL*-eGFP and NRL. Scale bar in H = 100 µm (applies to **A**–**H**); I = 50 µm.

*NRL*-eGFP expression was first observed in patches of cells in cultured OVs at d68 of differentiation (Fig. 3A,B), which increased dramatically over time (Fig. 3C–F). By d89, all OVs displayed robust *NRL*-eGFP expression (Fig. 3E,F) that persisted at least to d230, the latest time point tested (Fig. 2G,H). Outer segment-like protrusions from the surface of OVs were first noted at d140 (data not shown) and remained readily apparent by light microscopy until at least d230 (Fig. 3G,I). To determine the percentage of *NRL*-eGFP+ and NRL+ cells generated over time, we dissociated *NRL*^+/eGFP^ hESC-OVs at multiple stages of differentiation, immunostained for NRL, and quantified labelled cells using high content image analysis (HCIA). The percentage of *NRL*-eGFP+ cells increased between d70–130 from 0.3 ± 0.3% to 47.2 ± 3.3% (n = 6; mean ± standard deviation); similarly, the percentage of cells labeled with an NRL primary antibody increased from 5.7 ± 2.3% to 50.3 ± 1.9% (Fig. 3J). Within the d70–130 time window, the most dramatic increase in *NRL*-eGFP and NRL expression occurred between d70 and d95 (Fig. 3J), which ultimately led to approximately half of the entire cell population of NRL^+/eGFP^ hESC-OVs adopting an early rod identity. Of note, the difference between the percentage of cells at d70 expressing *NRL*-eGFP vs. NRL likely reflects a relative delay in visible expression of the transgene in live cultures.

### Specificity of eGFP expression during early rod production in *NRL*^+/eGFP^ OVs

**Figure 4:**
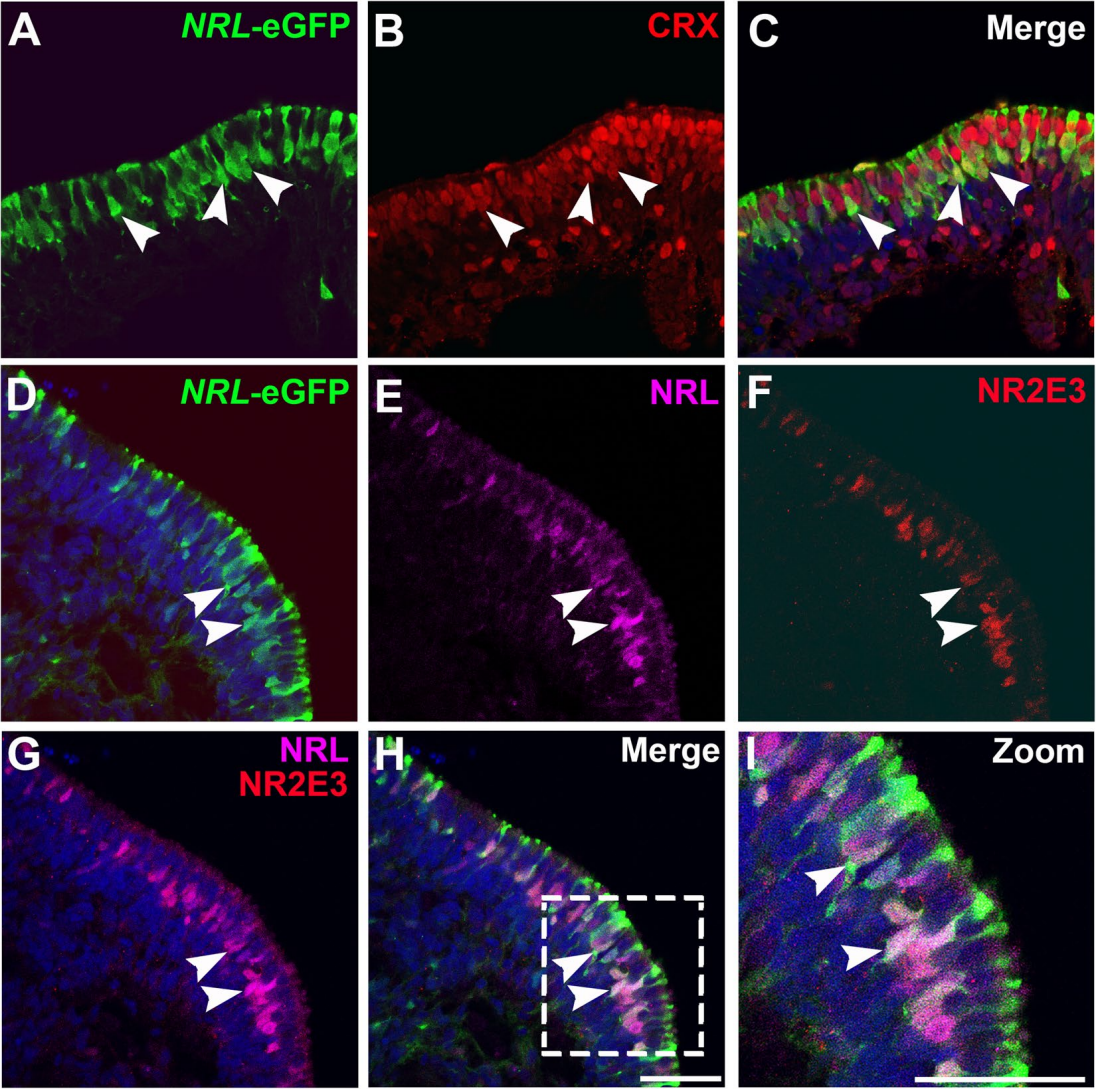
eGFP expression and specificity during early rod production in *NRL*^+/eGFP^ OVs. To examine early rod production in *NRL*^+/eGFP^ OVs, OVs at d90 of differentiation were analyzed by immunocytochemistry. (**A–C**). All *NRL*-eGFP+ cells co-expressed CRX (arrowheads), but not all CRX+ cells expressed eGFP. (**D–I**) *NRL*-eGFP (**D**) co-labeled with NRL (**E**) and NR2E3 (**F**), demonstrating specific expression of eGFP in rod photoreceptors (merged images in (**G**–**I**). Examples of co-localization of NRL and NR2E3 with eGFP highlighted by arrowheads in panels D–I. Scale bars = 50 µm.

To evaluate the specificity of expression of the *NRL*^+/eGFP^ line, we first compared localization of eGFP to that of the pan-photoreceptor transcription factor CRX (cone rod homeobox) at d90 of differentiation, a time point coinciding with early rod production (Fig. 4A–C). All eGFP+ cells had CRX+ nuclei and well-defined eGFP-labeled projections that extended to the OV surface (Fig. 4A–C). Most of the eGFP+/CRX+ cell bodies were located within a thin band that was separated from the OV surface by a similarly thin row of eGFP−/CRX+ cells. As expected, eGFP+ cells were also immunopositive for the rod-specific transcription factors NRL and NR2E3 (Fig. 4D–I). To confirm co-expression of eGFP and NRL, manual counts were performed on OV sections. At this stage, 95.7 ± 0.02% of eGFP+ cells had pronounced NRL expression (n = 3; mean ± standard deviation). Conversely, 98.8 ± 0.02% NRL+ cells also expressed eGFP. These results confirm that the *NRL*^+/eGFP^ line specifically and robustly labels early rods in OV cultures.

### eGFP labeling of rods in mid-stage *NRL*^+/eGFP^ OVs

**Figure 5:**
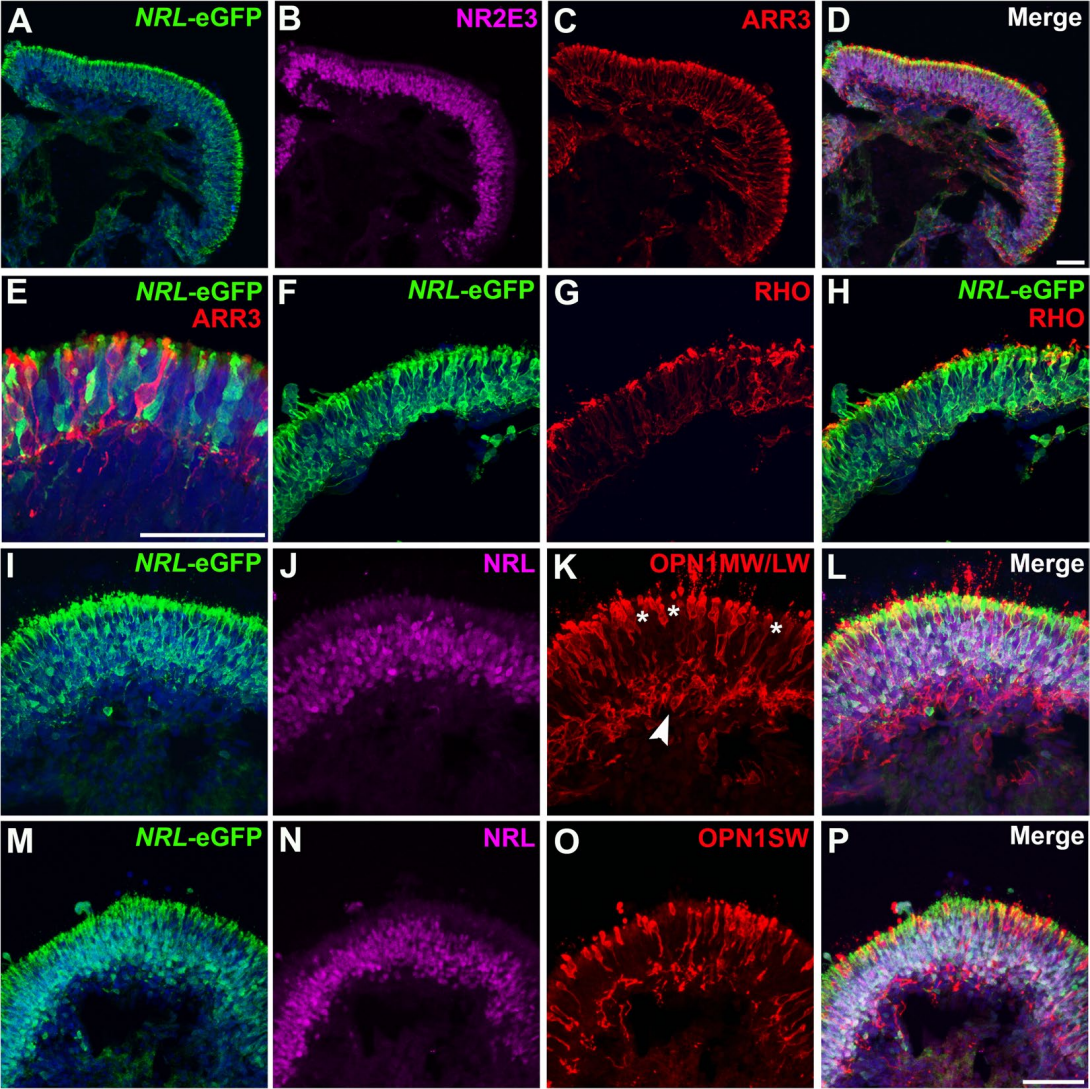
eGFP labeling of rods in mid-stage *NRL*^+/eGFP^ OVs. To examine mid-stages of rod differentiation in *NRL*^+/eGFP^ OVs, d145 OVs were analyzed. (**A,B**) A large increase in rod production was observed by d145, as shown by co-expression of *NRL*-eGFP+ (**A**) and NR2E3 (**B**). (**C**,**D**) Numerous ARR3+ cones were also present at this time (**C**; merged image in **D**). (**E**) Higher magnification image of showing non-overlapping *NRL*-eGFP and ARR3 labeling in rods and cones. (**F–H**) Many *NRL*-eGFP+ rods (**F**) also expressed rhodopsin (RHO) (**G**; merged image in **H**) at this stage of differentiation. (**I–P**) Analysis of *NRL*-eGFP and NRL expression relative to red/green cone opsin (OPN1MW/LW; **I**–**L**) or blue cone opsin (OPN1SW; **M**–**P**) expression showed complete segregation of all cone opsins from *NRL*-eGFP+/NRL+ cells. Note the predominant localization of cone opsin-expressing cells along the outer (apical) portion of OVs (asterisks in **K**), although some cones were also found beneath the organized photoreceptor outer nuclear layer (arrowhead in **K**). Scale bars = 50 µm.

Markedly increased eGFP expression was noted by d145 of OV differentiation (Fig. 5), coincident with an increase in the thickness of the NRL+ and NR2E3+ rod layer (6–8 nuclei) in the developing outer nuclear layer (ONL). All eGFP+ cells were immunopositive for NR2E3 and immunonegative for the cone-specific marker ARR3 (Fig. 5A–E). To further examine eGFP expression in maturing rods, rhodopsin (RHO) expression was analyzed via immunocytochemistry (ICC). At d145, RHO was expressed in some, but not all NRL+ rods, but all RHO+ cells co-expressed eGFP (Fig. 5F–H). Manual counts confirmed that all eGFP+ cells co-expressed NRL, while 98.8 ± 0.01% NRL+ cells co-expressed eGFP+ (n = 3 for each). RHO expression was more variable, with eGFP+ cells co-expressing RHO in 67.7 ± 0.24% of cells (n = 3). Cone opsin ICC was also performed on *NRL*^+/eGFP^ OV sections (Fig. 5I–P), with medium/long-wavelength cones (OPN1MW/OPN1LW) found primarily in a highly organized layer along the apical portion of OVs (Fig. 5K). OPN1MW/OPN1LW+ cones were also present deep to the eGFP+/NRL+ rod layer where they lacked the organized structure found in the overlying ONL (Fig. 5K,L). Similarly, short wavelength cones (OPN1SW) were found predominantly in the apical ONL (Fig. 5O,P). In all sections, eGFP co-localized with NRL and NR2E3 but not with cone opsins.

### eGFP labeling of rods in late stage *NRL*^+/*eGFP*^ OVs

**Figure 6:**
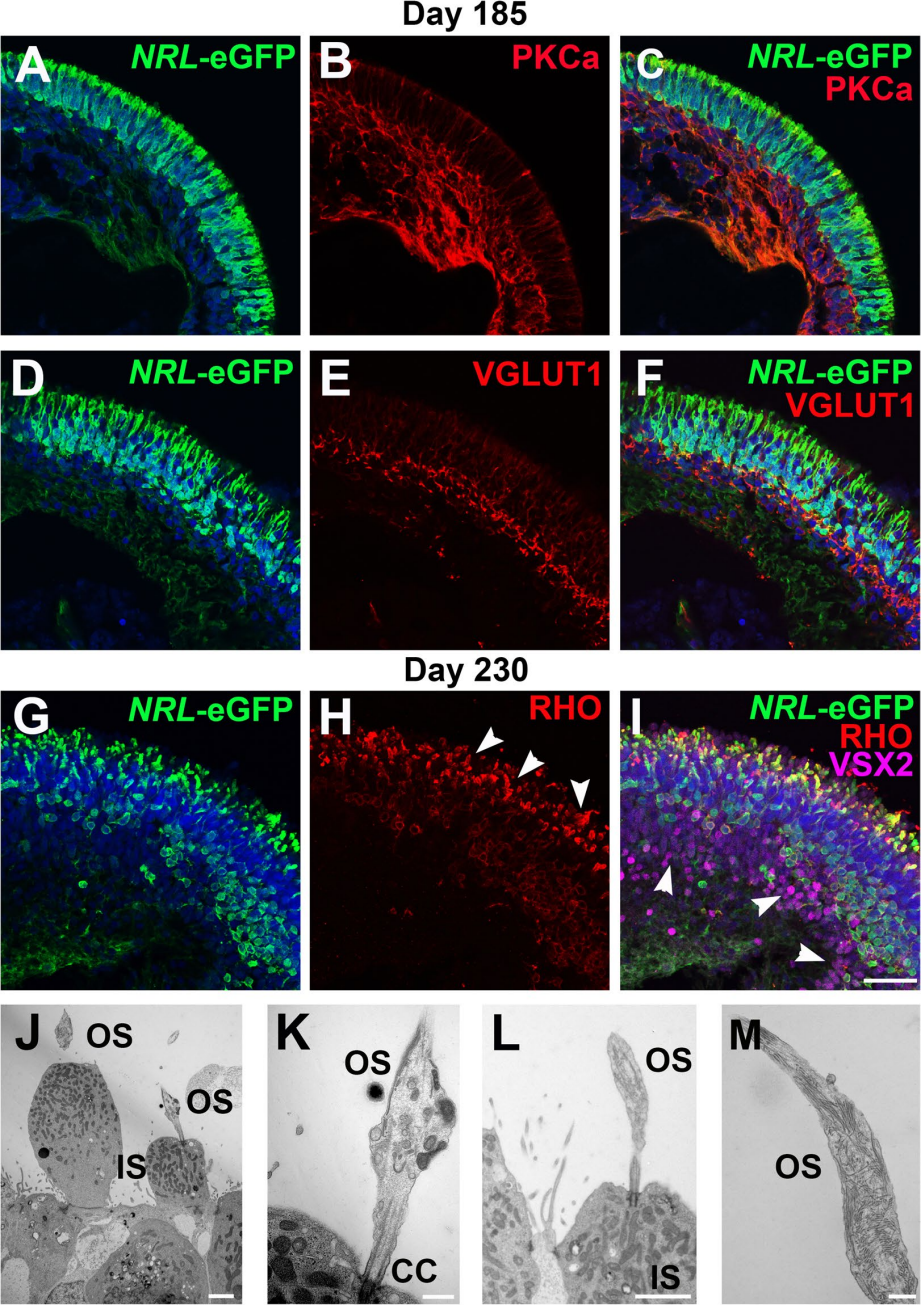
eGFP labeling of rods in mature *NRL*^+/eGFP^ OVs. To examine late stages of rod differentiation in *NRL*^+/eGFP^ OVs, d185 and d230 OVs were analyzed. (**A**–**C**) At d185, PKCα (a rod bipolar cell marker) was detected beneath the layer of *NRL*-eGFP+ rods, consistent with the development of an inner nuclear layer. (**D–F**) *NRL*-eGFP+ rod terminals co-labeled with the synaptic protein VGLUT1 within a discrete outer plexiform layer. (**G**–**I**) At d230, RHO immunolabeling was found in rod outer segment-like projections (arrowheads in **H**) that co-expressed *NRL*-eGFP (merged image in **I**). VSX2, a marker of rod and cone bipolar cells, was found beneath the layer of *NRL*-eGFP+ rods (arrowheads in **I**), demonstrating maintenance of an inner nuclear layer at late stages of differentiation. (**J**–**M**) Representative transmission electron micrographs of showing photoreceptor outer segments (OS) and mitochondria-rich photoreceptor inner segments (IS) linked by a connecting cilium (CC) along the outside of *NRL*^+/eGFP^ OVs at d230. Scale bar in I = 50 µm; J,L = 2 µm; K,M = 500 nm.

Lastly, eGFP fluorescence in *NRL*^+/*eGFP*^ OVs was examined relative to the expression of markers of retinal maturity at time points ≥d185 (Fig. 6). At day 185, 98.9 ± 0.01% of eGFP+ cells co-expressed NRL while 100% of NRL+ cells co-expressed eGFP (n = 4). Immunolabelling for the rod bipolar cell (BPC) marker PKCα (Fig. 6A–C) and the cone and rod BPC marker VSX2 marker (Fig. 6I) was found directly beneath the *NRL*-eGFP+ ONL, reminiscent of the inner nuclear layer (INL) observed *in vivo*. Of note, retinal ganglion cells (RGCs) were largely absent by this time in culture, possibly due to lack of diffusion and metabolic support to the innermost portion of the OV where RGCs reside (see Fig. 2H). To look for synapse formation and possible development of an outer plexiform layer (OPL) in *NRL*^+/eGFP^ OVs, VGLUT1 ICC was performed at d185. VGLUT1 labeling was found on eGFP+ rod terminals (Fig. 6D–F) within an OPL-like band between the ONL and INL. VGLUT1+ puncta were also present that did not co-localize with eGFP, consistent with its expression in both cone and rod terminals. At d230 of differentiation, RHO immunostaining was present primarily within elongated outer segments that extended from the OV surface, where it also co-localized with eGFP (Fig. 6G–I; compare to Fig. 5G). At this stage, 88.1 ± 0.11% (n = 5) of eGFP+ rods co-expressed RHO, and eGFP and NRL remained co-expressed in virtually all cells (percentage of cells eGFP+ that are also NRL+ = 99.6 ± 0.01%; percentage of NRL+ cells that are also eGFP+ = 98.1% ± 0.04%; n = 4). An INL was maintained at d230 as shown by the presence and location of VSX2 + BPCs (Fig. 6I). To confirm that the OV surface projections corresponded to outer segments, transmission electron microscopy was performed at d230. Mitochondria-rich inner segments were found along the OV periphery (Fig. 6J–L), many of which were attached to developing outer segments via a connecting cilium (Fig. J–M).

## Discussion

In this study, we produced the first rod-specific hPSC reporter line by knocking the *eGFP* transgene into an endogenous *NRL* locus, a strategy that increases the likelihood that reporter expression will faithfully mirror that of NRL. This targeted knock-in strategy is also novel among mammalian rod reporter lines, since the widely used *Nrl*-GFP mouse was created by inserting multiple copies of an *Nrl-GFP* transgene randomly in the murine genome^1^. While our approach does create a nonfunctional *NRL* allele, no phenotypic consequences are observed in human patients heterozygous for *NRL* loss of function mutations^29,30^.

The WA09 *NRL*^+/eGFP^ line strongly labels NRL+ rods throughout OV differentiation, demarcating the entire cell from axon terminal to outer segment. The first committed *NRL*-eGFP rods arose at d68 in differentiating OVs, consistent with the time course of human prenatal rod development^31^. Thereafter, the *NRL*^+/eGFP^ rod population expanded to account for the bulk of the OV cell population, with approximately half of all cells expressing eGFP and other rod markers by d130. Mature eGFP+ rods developed pronounced RHO + outer segments that populated the apical surface of OVs and became more prominent with time. These findings are consistent with previous reports of 3D retinal organoids produced from hPSCs^24–28^, and are indicative of a well-organized ONL. Unlabeled cones were also readily generated in *NRL*^+/eGFP^ OVs, typically near the apical surface of the ONL where they are also located *in vivo*. Rods produced in *NRL*^+/eGFP^ OVs possessed VGLUT1+ axon terminals that aligned themselves within an OPL-like layer, further demonstrating the capacity of *NRL*^+/eGFP^ OVs to generate polarized photoreceptor progeny within highly organized laminae. However, not all cells were correctly positioned within laminar structures, as occasional clusters of cone and rod photoreceptors were also present within the INL-like layer.

Given these findings, the *NRL*^+/eGFP^ reporter line has potential to play a valuable role in facilitating basic and translational studies using hPSC-OV technology. For example, the capacity to specifically label all rods provides a platform to study human rod development or test protocols that bias toward or against rod production relative to cones. In addition, mutations in genes known to cause primary rod dysfunction and death (e.g., retinitis pigmentosa) could be edited into the *NRL*^+/eGFP^ line, allowing rapid and accurate assessment of rod-specific outcomes relative to the unedited, isogenic *NRL*^+/eGFP^ line.

Access to an unlimited supply of fluorescently labeled, hPSC-derived rods may also accelerate the development of rod replacement strategies for patients with retinitis pigmentosa and other rod degenerative diseases. While labeled cells generated by reporter lines would presumably not be used directly in therapies, they could serve as a powerful tool for proof-of-concept studies and for tracking rod fate post-transplantation in animal models. Caution would need to be exercised in such preclinical studies to distinguish truly integrated donor cells from host cells taking up eGFP by biomaterial transfer^32–35^. Toward this end, the ability to use human-specific primary antibodies to identify hPSC-derived donor cells in xenografts would offer an added level of confidence when interpreting results.

## Methods

### Generation of the WA09 *NRL*^+/eGFP^ reporter line

The following fragments were amplified to create the donor plasmid for Cas9D10A-mediated homologous recombination: (1) an 800 bp fragment 5′ of the *NRL* start codon (amplified from WA09 hESC genomic DNA) engineered with flanking KpnI and SalI restriction enzyme sites, (2) an 837 bp fragment 3′ of the *NRL* start codon (amplified from WA09 hESC genomic DNA) engineered with flanking BamHI and NotI sites, and (3) *eGFP* coding sequence with the rabbit beta-globin polyA terminator flanked by SalI and MfeI sites (amplified from the plasmid AAVS1-Pur-CAG-EGFP; Addgene #80945). The three amplicons were ligated into the PL552 plasmid backbone (Addgene #68407) containing a floxed puromycin resistance (*PuroR*) gene expression cassette, and the SalI cloning site located between the ATG and the *eGFP* coding sequence was eliminated by site-directed mutagenesis using the NEB Q5 Site-directed Mutagenesis kit. The resulting plasmid (Fig. 1A) was fully sequenced to confirm correct assembly and sequence. Two sgRNAs targeting opposite strands flanking the *NRL* start codon (TGACATATTCCATGGCCAGG and GTAAAGCGGGAACCCTCTGA) were identified using the online crispr.mit.edu tool and cloned into the Cas9 sgRNA plasmid (Addgene #68463) according to the published protocol^36^. A full list of cloning primers can be found in Supplemental Table 1. For electroporation, WA09 hESC cells were cultured in hESC medium [DMEM/F12 (1:1), MEM non-essential amino acids, 0.5× GlutaMAX (Thermo Fisher), 0.1 mM β-mercaptoethanol (Sigma), and 4 ng/ml bFGF (R&D systems)] on mouse embryonic fibroblast feeder (MEF) cells with RHO kinase (ROCK) inhibitor (0.5 mM, Calbiochem, H-1152P) for 24 hours before being harvested with TrypLE Express enzyme (Thermo Fisher) and collected in DMEM/F12 (1:1) with ROCK inhibitor. 1 × 10^7^ cells were electroporated with a cocktail of 15 µg CAG-Cas9D10A plasmid (Addgene #44720), 15 µg of each sgRNA plasmid, and 30 µg donor plasmid in 500 µl of electroporation buffer (KCl 5 mM, MgCl2 5 mM, HEPES 15 mM, Na_2_HPO_4_ 102.94 mM, NaH_2_PO_4_ 47.06 mM, pH = 7.2) using the Gene Pulser Xcell System (Bio-Rad) at settings of 250 V and 500 μF in 0.4 cm cuvettes (Phenix Research Products). Following electroporation, cells were plated on MEF feeder cells in 0.5 μM ROCK inhibitor and 5 µM L-755,507, a β3-adrenergic receptor agonist that has been shown to bias cells toward homologous repair over non-homologous end joining^37^. At 72 hours post-electroporation, cells were treated with puromycin (0.5 µg/mL, Invivogen, ant-pr-1) in MEF-conditioned hESC media to select for cells incorporating the plasmid. Puromycin treatment was increased to 1 µg/mL on day (d) 16 post-electroporation and maintained at this level until colonies were large enough for passaging. Puromycin was removed and 0.5 µM ROCK inhibitor was added 24 hours prior to passaging and screening clones. Individual colonies were manually selected, genomic DNA was isolated from a portion of each colony using QuickExtract DNA Extraction Solution 1.0 (Epicentre), and the presence of a single insertion event at only one *NRL* locus was confirmed by PCR analysis (discussed in the Results section below). One clone was selected and the *NRL* loci were fully sequenced to confirm targeted insertion. In addition, the top ten predicted off-target sites (identified using the online crispr.mit.edu tool) for each sgRNA were PCR-amplified and sequenced to confirm that no non-targeted editing occurred. Genotyping, off-target primer sequences, off-target sequences, and off-target amplicons can be found in Supplemental Information.

### CRE/LoxP recombination

To enhance reporter gene expression, the floxed *PGK-PuroR* cassette was removed using CRE-mediated recombination. Briefly, hESCs were digested with TrypLE Express enzyme (Life Technologies) and harvested in 50 µl DMEM/F12 with 0.5 µM ROCK inhibitor. 10 µL of TAT-CRE recombinase (Excellgen) was then added to the cells and incubated for 20 minutes at 37 °C. Subsequently, cells were triturated to ensure single-cell dispersion and plated in 6-well plates for clonal selection in hESC media with 0.5 µM ROCK inhibitor. Individual colonies were manually selected and PCR verified for loxP recombination and precision removal of the *PuroR* cassette.

### Genotyping of *NRL*^+/eGFP^ OVs

Confirmation of targeted *eGFP* insertion at one *NRL* locus was also performed in differentiating *NRL*^+/eGFP^ optic vesicles (OVs, or retinal organoids). Genomic DNA was isolated from OVs using QuickExtract DNA extraction solution 1.0 (Epicentre). Amplification was carried out using 2 × Q5 PCR master mix (NEB) and two different primer pairs: (1) left junction primers (red arrows in Fig. 1B; forward: 5′-TGTTACAAAGTCTAACTGCCC-3′; reverse: 5′-GGTGGTGCAGATGAACTTCAGG-3′) that predict an amplicon of 986 bp for a targeted *NRL* allele and no amplification of an unedited, wildtype *NRL* allele, and 2) homology arm primers (blue arrows in Fig. 1B; forward: 5′-CACCATCCCTCTGGCTTTCC-3′; reverse: 5′-GCAGGGTAGCCAGCCAGTAC-3′) that predict (1) a sole amplicon of 295 bp in an unedited line, (2) two amplicons of 295 bp and 3.2 kb in a targeted *NRL*^+/eGFP^ clone retaining the *PuroR* cassette (*NRL*-*eGFP*+*PuroR*), and (3) two amplicons of 295 bp and 2.1 kb in a targeted *NRL*^+/eGFP^ clone following successful excision of the *PuroR* cassette (*NRL*-*eGFP*− *PuroR*). PCR cycling conditions were as follows: 30 s at 98 °C (initial denaturation), then 35 cycles of 10 s at 98 °C (denaturation), 20 s at 63 °C (annealing) and 60 s at 72 °C (extension), followed by 2 min at 72 °C (final extension), with the exception that the extension time was increased to 90 s for reactions using the homology arm primers. Note that the 3.2 kb fragment predicted for *NRL*-*eGFP*+*PuroR* clones does not amplify even with the increased extension time.

### hESC culture and retinal differentiation

Pluripotent hESC colonies were maintained in mTeSR1 on matrigel-coated plates. For retinal differentiation, hESCs were differentiated first to optic vesicle-like (OV) structures based upon previously described protocols^27,38–42^ with some modifications. Briefly, hESC colonies were enzymatically lifted and weaned into neural induction medium [NIM; DMEM/F12 1:1, 1% N2 supplement, MEM nonessential amino acids, 2 mM glutamax (ThermoFisher) and 2 µg/ml heparin sulfate] over the course of four days. On d6, aggregates were treated with 1.5 nM recombinant BMP4^43^ which was diluted every three days by 50% media changes over the course of nine days. On d7, suspended cell aggregates were re-attached to Matrigel-coated plates and on d16 cultures were switched to retinal differentiation medium [RDM; DMEM/F12 (3:1), 2% B27 supplement, MEM nonessential amino acids, 2 mM GlutaMAX and penicillin-streptomycin]. At d30, NR structures were manually dissected from the plate and allowed to form free-floating OVs, whereupon they were further differentiated in 3D-RDM [5% FBS, 3:1 DMEM/F12, 2 mM Glutamax, 2% B27 supplement, 200 µM taurine, 1:1000 chemically defined lipid concentrated (Thermo Fisher: 11905031), penicillin-streptomycin] supplemented with 1 µM retinoic acid until d120 of differentiation when the retinoic acid was removed. Live OVs were imaged on a Nikon Ts2-FL equipped with epifluorescence using a DS-Fi3 camera.

### Immunocytochemistry

*NRL*^+/eGFP^ OVs were processed for immunocytochemistry (ICC) as previously described^38,40–42^. Briefly, OVs were fixed in 4% paraformaldehyde for 35 min, washed in PBS, cryoprotected for 1 hr at RT in 15% sucrose followed by overnight at 4 °C in 30% sucrose, and cryosectioned. For immunolabeling, frozen sections were first blocked (10% NDS, 5% BSA, 1% fish gelatin, and 0.5% Triton-X in PBS) for 1 hr, followed by incubation with primary antibodies overnight at 4 °C (Supplemental Information). On d2, primary antibodies were removed with PBS washes and sections were incubated with secondary antibodies at 1:500 (Alexa Fluor, Thermo Fisher) for 30 min. After washing and mounting with Prolong Gold (Thermo Fisher), samples were imaged on an A1R-Si confocal microscope (Nikon). Manual counts were performed using single optical plane confocal micrographs to determine co-expression of eGFP, NRL, and RHO (n ≥ 3 per time point).

### High content imaging analysis (HCIA)

*NRL*^+/eGFP^ hESC-OVs were dissociated with papain (Worthington Biochemical Corp.) and plated on 96 well plates coated with poly-L-lysine at 40,000 cells/well. ICC was performed and cell counting was done using unbiased HCIA (Operetta, Perkin Elmer). 20 fields were captured in each well of a 96 well plate at 20X magnification (n = 6 per time point) and images were imported into Columbus software for analysis (Perkin Elmer). Pyknotic nuclei were excluded based on size, shape, and DAPI fluorescence intensity, as were all cells touching borders. Positive cells were determined by scatter plot gating of fluorescence intensity. Identical parameters for positive cell identification were used across samples for consistency.

### Transmission electron microscopy

Whole OVs were fixed overnight at 4 °C in 3% glutaraldehyde/1% paraformaldehyde in 0.08 M sodium cacodylate buffer, washed in 0.1 M cacodylate, and post-fixed in 1% osmium tetroxide in 0.1 M sodium cacodylate for 2 hrs at RT. Samples were subsequently dehydrated in a graded ethanol series, further dehydrated in propylene oxide, and embedded in Epon eposy resin. Semi-thin (1 µm) sections were cut with a Leica EM UC6 Ultramicrotome and examined under a light microscope to establish proper orientation. Ultra-thin sections were cut with the same microtome and collected on pioloform-coated 1 hole slot grids (Ted Pella Inc, cat # 19244). Sections were contrasted with Reynolds lead citrate and 8% uranyl acetate in 50% EtOH. Ultrathin sections were imaged with a Philips CM120 electron microscope equipped with an AMT BioSprint side mounted digital camera using AMT Capture Engine software.

### Data availability statement

The datasets generated during and/or analyzed during the current study are available from the corresponding author on reasonable request.

## Electronic supplementary material

Supplementary Information
