# Supplementary material for: Generation of a rod-specific NRL reporter line in human pluripotent stem cells
Source: Sci Rep. 2018 Feb 5;8:2370. doi: 10.1038/s41598-018-20813-3 (PMC5799252; doi:10.1038/s41598-018-20813-3)
Supplement: Supplementary file 1 — Supplementary Information [file 41598_2018_20813_MOESM1_ESM.pdf]

Supplemental Information

Title

Generation of a rod-specific NRL reporter line in human pluripotent stem cells.

Authors/Affiliations

M. Joseph Phillips[1, 2,+], Elizabeth E. Capowski[1,+], Andrew Petersen[1], Alex D. Jansen[1], Katherine Barlow[1], Kimberly L Edwards[1], David M. Gamm[1,2,3,*]

1. Waisman Center, University of Wisconsin-Madison, Madison WI, USA

2. McPherson Eye Research Institute, University of Wisconsin-Madison, Madison WI, USA

3. Department of Ophthalmology and Visual Sciences, University of Wisconsin-Madison, Madison WI, USA

[+]MJP and EEC contributed equally to this work and are co-first authors

*Corresponding author

Supplemental Table 1. Primers.

**Cloning primers**	
NRL 5' arm F -KpnI	GAGAGGTACCAAGGGAAAACGGGTCAGAAGGG
NRL 5' arm R-SalI	GAGAGTCGACTCTGGAGCTGGGCTGGGAGG
NRL 3' arm F-BamHI	GAGAGGATCCCCCTCTGAGGGCCGACCTGG
NRL 3' arm R-NotI	GAGAGCGGCCGCGCTGCGTTTCCCTGTCCTGA
eGFP-SalI F	GAGAGTCGACATGGTGAGCAAGGGCGAGGAG
eGFP-polyA-MfeI R	GAGACAATTGGGAGATCCCTCGACCTGCAG
**Site directed mutagenesis**	
SDM-F	ATGGTGAGCAAGGGCGAG
SDM-R	TCTGGAGCTGGGCTGGGA
gRNAs	
NRL sgRNA 1A	TGACATATTCCATGGCCAGG
NRL sgRNA 1B	GTAAAGCGGGAACCCTCTGA
**Genotyping primers**	
NRL genotyping -F	TGTTACAAAGTCTAACTGCCC
eGFP-R	GGTGGTGCAGATGAACTTCAGG
NRL 5'-F	CACCATCCCTCTGGCTTTCC
NRL 3'-R	GCAGGGTAGCCAGCCAGTAC
**Copy number qPCR primers**	
eGFP-F	AGCTAAACGGCCACAAGTTC
eGFP-R	AAGTCGTGCTGCTTCATGTG
NRL exon 3 qPCR-F	TGTCACCCTGAGCAAATCAG
NRL exon 3 qPCR-R	TTGTCTTGGGGACTTCTTGG
**Off target primers**	
NRL sgRNA 1A OT1 F	TCGCCATCCAGTACAGATCC
NRL sgRNA 1A OT1 R	CCAGAAACAGGAACGCACGA

NRL sgRNA 1A OT3 F	ATTCGTGGTGTCCAGAGGCTA
NRL sgRNA 1A OT3 R	TGGCATCGTAAGTGCAAATGA
NRL sgRNA 1A OT4 F	AGAAGAATGCCCACGTCCTG
NRL sgRNA 1A OT4 R	CCCTCTCCAGAGTCCTCGAA
NRL sgRNA 1A OT5 F	ACCGGGTGCTCCCAGAATTA
NRL sgRNA 1A OT5 R	GCCAACACCGACAAGTTTGG
NRL sgRNA 1A OT7 F	ACATTGCTGTGGGCGATACA
NRL sgRNA 1A OT7 R	GAGAATTGGGAGCAGTGCCT
NRL sgRNA 1B OT1 F	AGGTCAGCCTCTTCAGCGAT
NRL sgRNA 1B OT1 R	TAATTCGGTCCAGACCTCGC
NRL sgRNA 1B OT2 F	GAATGGTTCCATCAGGCCCA
NRL sgRNA 1B OT2 R	TCCCAAGTTGCTGACATCCC
NRL sgRNA 1B OT3 F	GGAAATCTAAGGGTACTGTAGGG
NRL sgRNA 1B OT3 R	CAACCCACGGAAAGACACATC
NRL sgRNA 1B OT4 F	GCCTTAGCCAATTTGCTCATAAATC
NRL sgRNA 1B OT4 R	AGGATGTTGGCCTTTGCAGC
NRL sgRNA 1B OT5 F	TCCGTTCCATCTTTTGCGGA
NRL sgRNA 1B OT5 R	AGGGGTCAGTCAGTACGTCA
NRL sgRNA 1B OT6 F	CTCTGCTAGCCACCGTTCTC
NRL sgRNA 1B OT6 R	GTTTGGAGGATGGGGTCTGG
NRL sgRNA 1B OT7 F	GGTGTCAGGAGAGCTTGTGG
NRL sgRNA 1B OT7 R	GAAGTTGCGACACAGAGGGA
NRL sgRNA 1B OT8 F	GAGTTGCCTGGAGGAAGCTAA
NRL sgRNA 1B OT8 R	AGTTTGCAATACTCTCTCAAAAGCA
NRL sgRNA 1B OT9 F	AGCTGAGGAGGGTACTGAGG
NRL sgRNA 1B OT9 R	ACACTCTGCTGTCCTGCTTC
NRL sgRNA 1B OT10 F	CCTTCAATGCCTCTCCGTGT

NRL sgRNA 1B OT10 R	CTTTTGGCCTTGCTGTGACC

Supplemental Table 2. Off target sequences.

sgRNA1A							
chromosome	strand	position	sequence	n_mismatches	score	ontarget	gene
chr14	1	24552017	TGACATATTCCATGGCCAGGGGG	0	100	True	NM_006177
chr20	1	39317426	TGACATACTCCATGGCCAGCGGG	2	4.214362	False	NM_005461
chr5	1	26555853	TCTCATATTCTATGGCCAGGGAG	3	1.500342	False	None
chr15	1	99428977	TGAAATATTGTATGGCCAGGTGG	3	1.332228	False	None
chr6	-1	1.36E+08	AGAGATATTCCAAGGCCAGGCAG	3	1.150704	False	None
chr6	1	12088422	TTACATCTTCCATGGCCAGTGAG	3	1.019098	False	None
chr15	-1	99563969	TGACAACCTCCATGGCCAGGTAG	3	0.958618	False	None
chr2	1	1.4E+08	TGTCATATTTCATGGCCAAGGAG	3	0.958556	False	None
chr3	-1	46932745	TCACCTTCTCCATGGCCAGGAGG	4	0.932256	False	None
chr17	-1	74383417	TGCCCTTCTCCATGGCCAGGAAG	4	0.905329	False	NM_001142601
chr16	-1	68573852	TGGGGTTTTCCATGGCCAGGAGG	4	0.891867	False	None
sgRNA1B							
chromosome	strand	position	sequence	n_mismatches	score	ontarget	gene
chr14	-1	24551968	GTAAAGCGGGAACCCTCTGAGGG	0	100	True	NM_006177
chr8	-1	22398127	CTGAAGCGAGAACCCTCTGATGG	3	1.609912	False	NM_001243974
chr11	1	1.13E+08	CTATTGCTGGAACCCTCTGATAG	4	1.386187	False	None
chr3	1	72786510	GAGAGGCAGGAACCCTCTGAAGG	4	1.345833	False	None
chr10	1	92490398	CTAAAGAGGGAACCCTCTGGGAG	3	1.054856	False	None
chrX	-1	1.5E+08	GGAAAGTGGGAACCCTCTGGAAG	3	1.019098	False	None
chr1	1	6113546	GCCGAGGGGGAACCCTCTGAGAG	4	0.905329	False	None
chrX	1	55306856	CGAAAACTGGAACCCTCTGAGAG	4	0.838643	False	None
chr6	1	62641171	ATAAATCCGTAACCCTCTGAAGG	4	0.797206	False	None
chr10	1	19062244	GTCAAGCCTCAACCCTCTGACAG	4	0.76913	False	None
chr8	-1	52335592	ATATAGCTGGAACCCTCTGCAGG	4	0.710795	False	None

Supplemental Table 3. Primary antibodies used for immunocytochemistry.

Antibody	Host	Catalog #	Company
ARR3	Goat	NBP1-37003	Novus
CRX	Mouse	h00001406-m02	Abnova
eGFP	Mouse	MB3580	Millipore
eGFP	Rabbit	A11122	Thermo Fisher Scientific
KI67	Rabbit	sc-15402	Santa Cruz BioTechnology
NR2E3	Mouse	ab41922	AbCam
NRL	Goat	AF2945	R&D
OPN1MW/LW	Rabbit	AB5405	Millipore
OPN1SW	Rabbit	AB5407	Millipore
PKCα	Rabbit	AB32376	Abcam
RHO	Mouse	MABN5356	Millipore
SNCG	Mouse	H00006623-M01	Abnova
VGLUT1	Guinea Pig	AB5905	Millipore
VSX2	Sheep	X1179P	Exalpha

Full-length gel from Figure 1, panels C and D.

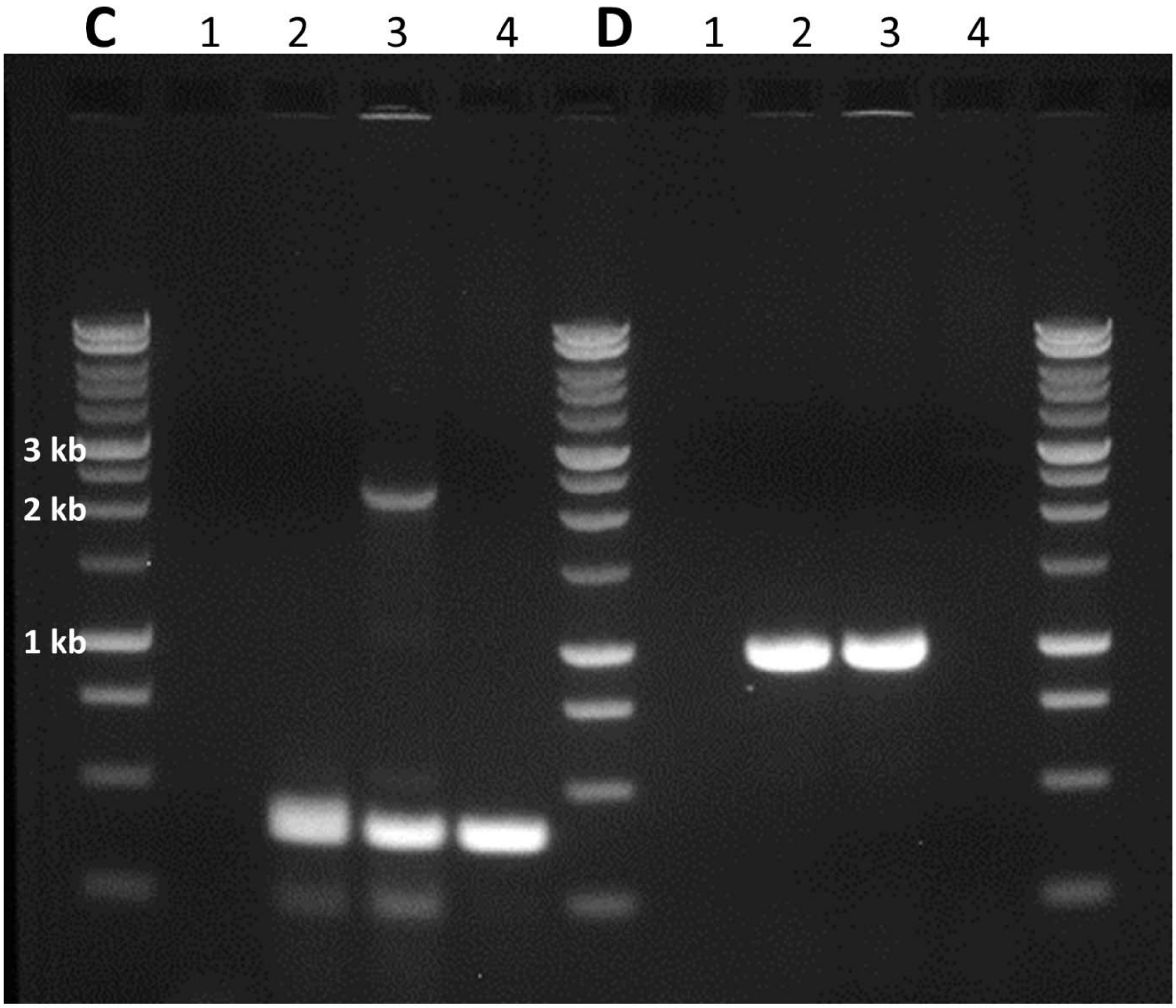

Off target amplicons for sgRNA 1A and sgRNA 1B. 10 off target sites for each sgRNA were sequenced and no indels were detected. The off-target sequence is noted with brackets.

sgRNA 1A

Off target site 1: NC_000020.11: 40688583-40689751 Homo sapiens chromosome 20, GRCh38.p7 Primary Assembly

TCGCCATCCAGTACAGATCCTCGAGGTGTGTCTTCTGTTCGGTCGGGCTGAAGCTGGGCGACGAGGGCACGGAGC
TACACGGAGTGCTGAGCGGTGTGGAGGACACCGAGCCGGCTGGCTGCAGGCGTGTGCAGGGCCTGCCCGGACG
CTCCGCGCGCCCCAGTGGCTCCTTCTTCACGTCGAACTTGAGCAGGTCGAAGTCGT[TGACATACTCCATGGCCAGC
GGG]CTGGTGGGCAGCTCTGGCCCCATGCTCAGCTCCGCGGCCATCGCTGAAGCGAGGCGCAGCCGCCGCTGCCG
CCCGGGAAACTTTGCGGCCGGCCGGAGCGCGCCGAGCCAAGCGCGGGGGGGAAGAGCGGAGAAGAGCTGGGG
AGGCGGGGAGCGAGGGCGCAGCGGGCCGGGGCCGCCGGCCAAGCCTTTGTCTGGGGACGCGGCGGCGCGCCG
GAGAGTCCCGAGGCTGCCTGCACCGCCCCAGAGCTCTGGGCTGTGCCCGCGCAGGGACCGGGCCGGGTAGAGTC
GGGCGGGGTGGAGAGGCAAGCGGAGCGCGCGGTGGGGCTGAGGGGAGGCGTGGGGCGAGTGCCCGTTGCTCG
CTCTCTAGCTCTCTTGCTCTTACGCTCTCTCGCTCGCAGCCGCTCGCAGCTCGGCGGTGCAGCTGTGCTGGATCCGG
CGGCGCCGCAGCCTTTTATCGCCTCCTGATGTCACTGGGGTGCGGGGGCCCGGGCGGCCCGGTGCGCGGGCCAA
TAGCTGCACGGCCTCCGCGGCCCAGCGGCGCAGGGCGGGGCGCGCCTGACAGCTCCCCCGCCCCCCGCGTCAGCT
GACTGGCGGCCCGAGCGGCCCCGGAGCGGCGGAGGCCTGGCGGAGCGCTGGAGCGGAGTGGGACGGCCAGCC
TGGGCCCACCCCCGTACCCTGCAGGTCCCGGCCCACGCACGCTCGCCTGGAGTGCGCGCCCCACCTCTAGGCCAA
ATCACCGCTTTCCCCTCCTCGCGCACTCTCCTCCCTCAGTTCCCTTTGCACCCCACCCCCATCCCGTGTCACCCCCAAG
GAGGCTCAGAATGAGCGCCGGGACAACGCCTCCTGGGCCCTTTGTTCCCAAGCGGCCCCCGCCCAGTGGGCGACG
CTCTGTGTGTCCTCGCGGCTTCTGGCCGTGTGTGTCGTGCGTTCCTGTTTCTGG

sgRNA 1A

Off target site 2: NC_000005.10: 26555045-26556014 Homo sapiens chromosome 5, GRCh38.p7 Primary Assembly

ATTCGTGGTGTCCAGAGGCTAGCAGTTCTGGTAAGAATAAAGTAGTTTCTCCTTATCCATGAAGGATACATTCCAA
GATCACCAGTGGATGCTTGAAGCCATGGGGAGTACAAAACTATGTACATCATGATTTTTCCTATAAATACATACCT
ATAATAATGTTTAATTGATAAATTAGGAACATTAAGATATTTACAATAACTAATAATAAAATAGAACAATATAACAA
TAGCCTATAATAGTATTTGTGTTCATATAGTCTCTCTCTCAAAATATCTTGTACTGTGCTGATTTATTTTTGCACCACA
CTTGACCATGGGTAACTGCAAAACTGTGGATAAAGGTGGAGTACTGCATTCTTAATTTGAAAATGTATTTCATATA
AATGCATAGAGTAATTTAAACAACTAGTATTATTAAAACTAAGCTAACAATTTTTCTTTCAAAAAAAGCATCAGCAC
AGAAACAACTGAAATTTTTGAAAACTAATTCAATTTGTGCTATTATATACAGTATTTACTGGATGTACACACGTGTG
GATGCGCACATGTGCACACACAAACACACACATGGGAAATCTTCCACATTACAAAAAGGAATATCAAGAGCCCATT
GGAGCACTTGATCTTTGATGACATCAACCTCTTCGGCTAATAATTTTCTCCAGAAAGGTCTTGACTTTCTGATTTCTT
TTTTTGTT[TCTCATATTCTATGGCCAGGGAG]TCTCACTCTCTAACTCCATTTTTATTTTCTAATTCTGTTGCATTGAA
TGTCTGTTCAATAAGTGTGTTCTCCGGGTTCATATCATTGGTTCTCTTCTCTCAAGCTTCTATGGCTCTGAGATAGAA
TATATTTCTTCCACATCTGTGGTCTCATAGAATAGTAAAAATAACTCTCCTGCAATCCTTACTAAGAATAATGTCCAT
GTTTATTTTCACTTTTAGGCATATCATTTGCACTTACGATGCCA

sgRNA 1A

Off target site 3: NC_000015.10: 98885303-98886249 Homo sapiens chromosome 15, GRCh38.p7 Primary Assembly

AGAAGAATGCCCACGTCCTGGGTGGACTTCTGTTCGGCCTGGGGCAGGACGCTGCATGTCAGTTCACCTAGAGCC
CCTCGCTTGCCCTCACCCCTCTCCCTTTCCTTCTCCTCCCCGTGTTGTCTCATGGTCAGTTCCTCTCCTGGCAAGGCAA
GAGAATCATAAAATCTCTTTGGACCATGTGTCTCTCTGATGCACTTTGGAGGTTGAAGCTCACTTTCGTGAAGTTAG
GAGCGGTTAATCCTCCTGCACGAGGATGCATGCCTGCTAGGGAAAGCCTCCCTGCGTTTATATGGAACAGTGGTG
TGTACCTTAGGGCAACCCAGAAACAGCTGAGATGGCCCTAAACTCTTCCGTATTTTGATTCAGAAGCTTTACCTTAT
TTGAACATCTGCAGAATAAGTTCTTTCCTTTAGAAATGATTGGGGATAAAATTGTTTGCATTA[TGAAATATTGTAT
GGCCAGGTGG]CCCCTCTTTCATGTTTTGTTCCTTGTGAAGAGGTTGAGTCTCCTATTTTTTTTTTTTTTTTGGAGATA
GAGTTTTGTTCTTGTCCCTCAGGCTGGAGTAAAATGGTGCAATCTTGGCTCACTGCAACCTCCGCCTCCTGGGTTCA
AACGATTCTCCTGCCTCAGCCTCCCAAGTAGCTGGGATCACAGGCACGTGCCACCACACCTGGCTAATTTTTGTATT
TTTAGTAAGAGATGGGGTTTCGCCATGTTGGGCAGGCTGGTCTTGAACTCCTGACCTCAGTTGATCTGCCCGCCTC
GGCCTCCCAAAGTGCTGGGATCACAGGCGTGAGCCACCACGCCCAGCCTATGGTATGCTTTTAAAAATGGTTTCTC
TGCACTAATGATTATTTAAAAAAAAAACTCTGGCAAGAACAAAAACAAATGGTTTTTTAAAAACTCTAGAAATAAT
TTGATACATTCGAGGACTCTGGAGAGGG

sgRNA 1A

Off target site 4: NC_000006.12: 136010901-136011617 Homo sapiens chromosome 6, GRCh38.p7 Primary Assembly

ACCGGGTGCTCCCAGAATTACTGCAAGGGAGGTTCATTCACCTGGGATTATACTTCATGCCGTGTGGGAAATTACA
TGCTCTCTAGTCAACTGTTGACATTACCAAGACCCTCAGCCTTGGAGATTTACATTTTACTCACACCTCAGACATAG
ACACAAGCTGGGGTTCATTTGTTTGTTTTACTTTTTCAGATGTTGGATGAAAAAAGAGATTCTTAATAATGGGTTTT
CACTTCTAACTCCCCCTTTCTCCCCATCAAAAAAACAAAACAAAACAAAACAAACCTCAAAAGACTTTATTTTCCTTT
CCCTTTCTTTATAGCAGAGATCAGGGTATCAGGGTATCAAGAGAGCAGGGTATCAGGCTGCTCTCTTATCCAAATG
GCAATACTCAAAGGTTCCCAAGATTTAGTCACCTCCCTTCTCAAGTGAATATAAAACGTGGAGCATCTTTCTTTGCA
GAGTAAGACTGACTTTAGGTCATCTTGAGCCCATTTATCATTATTTGAAACATTATAGAATCTATTGGATCATATAG
CCAAAGTTATAGAAAATATAATTTTCATTTTTAAACAGATCTCTTATTATGAATGGTTACTTGGG[CTGCCTGGCCTT
GGAATATCTCT]TTTGCATGATTGCAATATACTTGTGTCCAAATGTGGATCTTGGCCTCTTAAGTAATGTTCTCACAT
TTTACCCAAACTTGTCGGTGTTGGC

sgRNA 1A

Off target site 5: NC_000006.12:   12087892-12088555 Homo sapiens chromosome 6, GRCh38.p7 Primary Assembly

ACATTGCTGTGGGCGATACATTTATCAGGATGAAAATTTAGTTATTTTTGACTATTGTTGAGATGTAATG

TCCTATATTTCAAACTAGCTTGCTTCCTTTGTTCTGTTCTCCCACTTTGCCTTGGCAAGTTGGATCAATATGCAATTG
AAAGACGTTTGATCTGTAAATAGAAGGTTCTCTGAATTTCTTGGCCTTTCTGGCAAGATAGGGAGAGAGGCAGGT
AGATCAGAGTTTGAAGGGCCCATGGTGTGCGGTGTCTGCCTTTTATTTATTTGGAGTAGGATACCATATTGGAGG[
TTACATCTTCCATGGCCAGTGAG]CAGCGGTAGGGTCAGCATTGGAGACAACTTCTGAGCTGATGCGTTTATTTAA
ATTGTACTTACACATTTGGGATATGTAGAATGTTATATAATTATCAATATTGTCTTCAGTTCAGTACAAAATAGTACC
CTGGGAATTTGTTCCTTTTGCCATTTTAATAAGTACCTAGACCAGTGTGTTAAATTTGGAATCGATTACTGCCCATGT
CATGAAGTATGTATATCCTTATAAAGTTTAAATTAATTATTATTGAAATAGAGCATGCACATATTTACAAAATCCAG
TTGTGCCCAGAGCCCCAGGTCAAAGAACAGCAGGAGCAAGGCACTGCTCCCAATTCTC

sgRNA 1A

Off target site 6: NC_000015.10: 99020565..99021246 Homo sapiens chromosome 15, GRCh38.p7 Primary Assembly

GTGTCTGTAGGGGAACGTGGACCCAGCACGTCTGGGATTTCAGTTCTTGGGGAGCAGGGTCTCGCTGGGGACCAT
GGGCAGGGACTGGGCTGGATATTTGGACTCATAACACTGCCACAGACTATTCCCATTCCTTCATCAGTGTGAAAGG
AAAGGATGAGGTGGCCTCTGAAAGT[CTACCTGGCCATGGAGGTTGTCA]GTTACTTCTCACCCAAGCAGATAGTA
TAGTCTATCTGCTGTAGGAAAGTTTGCTAGGAGGGATTTTTTTGTGATTGGGTGTATCGACGAGCTGGCTTGCCAC
CACCTGGCCAGGGCTCTGGGCAGCCCATCCCCTAGAATGTCTTTCCAGGGGTCCTGTTCTTCCTGGGAGGGCCGG
GTCCACAGTGAGTTCCCCTGATGGCCAAGGCGTCTGTATTTCCATCTTCTGTTGTGATTCCTTGCCATGTAGTGGCA
TCTACACTAGAGATGGCTCTTATCACCCCCTTTCTACACATCAGGGGCTGTAGGATATCTGTATGAAATGGGATCTC
CACAGCATAAGATGGCTGGTGTCCTGTCCCTCTCCACATGGGTCCTGCCATGGCCTGGCAGGACAGAGTGAATGA
CCAGACTTGGATAGGGTGGTGGCAGGGCAGTGGATGCTGAGTGTTGTCGTTTTGAACTTTGAAGCCCTAGTCTGG
CG

sgRNA 1A

Off target site 7: NC_000002.12: 139531402-139532229 Homo sapiens chromosome 2, GRCh38.p7 Primary Assembly

ACAGAATGACTCAGCACCGTTTATGAATGATTATAATGAGTTGGGGTAAAGAGAGGTAACAGACATGGGGAAAA
AGATGACACACGATGATAAGAAGTGCAAGCAGCATTCTAAAATTCAGTTCACATCTTCAAACTCTGAGTTCAAGAT
GCTTTCCACTGTACCATGTGGTCCCTATCACTGAGTTCTATAGCATGTTTTCTATGTTTTAAAGCAAAAAGTTTCTCT
GGTTTGTCCTCCTCAAATTGTCCAACAAAAATATATATTGAAAACTCTCAGATTTGCTACACATCACTTGTGAAAAA
TTGCTTAAGAAATAATTCCTCAACTCATTCAGTTTCATTAATTTTGTCCTTTGAAGGATGGAAGAATATATCTTTCTA
GCAAAAATCGGATTTGTGCATGACAGCTACTTGAGCTCAGAGGTCAAAGAATGAATTTGGGCTGCCTCCCTGCATT
CCCATCCCTTCAATCTTTTGTTGTGCTTCCACAGCAGGTAGGATTTGAAAGAGATTCTGCCCTTGCTAGAAGAAAAA
AAAATTGTAAGGTAGAAAATAATCAAACCTTGGCTTGAGAGGAGCCAAAATGAATGCCGCTTCCAATACTAATGT
AAACCTTCTACCAATACTTAGGATTTACAAAAAAATTTCAAAAG[TGTCATATTTCATGGCCAAGGAG]ACCTAGTAT
GGTGGCGATGTATGCTATGCTTATGCCTGGGAATAGAGTAAGAGGTTAGGAAGATCAGGAGATAAATTTTCAAG
GAAAAAAATCTGAAAAAAGATTGGCATTTCCACAAATGTGTGAAAGGGAATGCAGACAGATAAACCT

sgRNA 1A

Off target site 8: NC_000003.12: 46891087-46892009 Homo sapiens chromosome 3, GRCh38.p7 Primary Assembly

CTTGTTGCAATGGCTGTGCTAACAGATAAATAGGTCCTAAAGTTCAGGGAGGCCAGAGGTGGGGCCACCACCCCT
ATGGATGAATGAAGGAGTGTGGGAGTCATGCGTGCTGGAAGCACATATCTTTCCCTCTCCTGAACATCCCAACTCC
AGAAAAAGGGGTGTGCCT[CCTCCTGGCCATGGAGAAGGTGA]AGCCAACAGCTGGAGGTAGAGGCTGAGCCAAT
TCCTTGAGCCAGTGAGGGGTGTGTTGGTCAGCTTTCGGTGGTCAGGCTGCCCAATGGCCACTCAGTTTTCAACCCA
TGACTGCCTGCCTGCAGTCTAGGCCATCAGGGGTGACCCCCAGTCATATCAGTAGGCTGGTCTGTCTGTTGGGAG
GCCCAGGGAGTCAGCAAGTCTCTAAGGATGTGTGGGAGCCTCCGTCAGTCACTCCAGAGCTCTCCATCAGCTGGA
GAAAATACAGCACAATGGGTTTGTCTTGAGAGGGGTGGAGGTGGCAGAAGAAGAAGGATAGTGGGGAGGGGG
TAAGACAACAAGATGGCAAAGGCAGTGTAGATGGTAGTTGTTGGGCAGGTGGCAGTATTGCTGATGGTGCTAAT
GTTGCCTTGGTGATGCTGGAGGTGGTGGCAGTGGTGCTGGTGCTGGTGATGTACCAGTTGTGATGGTGCAGATG
GTGGTGATGGTGGTTATATGGATGGTTACAGTAGTGCTCATGGTGGTAACGGTGATGCCAGTGGTGGTGGTGGT
AGTACTGGGAGGGACTGGTGATGCTGGTGTTGGTGTTAGTGACAGTAATGATGGTTATTGATGGAGGTACTAGCA
GCAACGATGGAAATGGTAGTTAATGGGTGCTGTAAATGGAGTGAGGTGGACAGGGATCAGCCCCCTCACCAGCA
TGGATCCCCCATGTCAGAGGCACAGGGAGA

sgRNA 1A

Off target site 9: NC_000017.11: 76386803-76387585 Homo sapiens chromosome 17, GRCh38.p7 Primary Assembly

CAGCTATGAGCAGGTCACCAATGAAGACCTCCTGACCAACTGCACGCTATTGCTGTGCCGCCGGCTGCTGTCACCC
ATGAACCTGCTGTCTCTGCACACGGCTTCGGGGCTGCGCCTCTTCTCTGTGCTCAGCCTGGCCTGGGGCTTCATTGC
TGATGTGGACCTAGAGAGTGAGAAGTATCGGCGTCTGGGGGAGATGCGCTTCACTCTGGGCACCTTCCTGCGTCT
GGCAGCCCTGCGCACCTACCGCGGCCGACTGGCCTACCTCCCTGTAGGAAGAGTGGGTTCCAAGACACCTGCCTC
CCCCGTTGTGGTCCAGCAGGGCCCGGTAGATGCACACCTTGTGCCACTGGAGGAGCCAGTGCCCTCTCACTGGAC
AGTGGTGCCCGACGAGGACTTTGTGCTAGTCCTGGCACTGCTGCACTCGCACCTGGGCAGTGAGATGTTTGCTGC
ACCCATGGGCCGCTGTGCAGCTGGCGTCATGCATCTGTTCTACGTGCGGGCGGGAGTGTCTCGTGCCATGCTGCT
GCGCCT[CTTCCTGGCCATGGAGAAGGGCA]GGCATATGGAGTATGAATGCCCCTACTTGGTATATGTGCCCGTGG
TCGCCTTCCGCTTGGAGCCCAAGGATGGGAAAGGTGTGTTTGCAGTGGATGGGGAATTGATGGTTAGCGAGGCC
GTGCAGGGCCAGGTGCACCCAAACTACTTCTGGATGGTCAGCGGTTGCGTGGAGCCCCCGCCCAGCTGGAAGCCC
CAGCAGATGCCACCGCCAGAAGAGCCCTTATG

sgRNA 1A

Off target site 10: NC_000016.10: 68539589..68540304 Homo sapiens chromosome 16, GRCh38.p7 Primary Assembly

CCGCTGAGCCTTTCATTTCGGGGATGGGGAGCGGCAGGCCCGGGCTGGTTCCACGGTCCTCGCCACCATCTCCCCT
GGAGATGTGGTTTAGGGGCGGAGGTGGGGCGGGGCGGGGCGGGGTGGGGTCGGTGTTGCAGCGGGGTGAGT
GGGCCCTGTCCTTTCTCCCCAGCTCCTGCCCCGGAGCCGGGCCCTGGCGAGGCAGGAATGGCCCCGAGGCCTCCG
ACCGCCGCGCCCCAGGTGAGCAACGCGTTCCTAACCTCCTGGGCATCCCATCCATCTATCCATCCCATCTCCCAAGG
GTGTTTGGAGCAGTCATTGTTCTCTGCCTGGCGACTCCCTTACTTGCTTGGCTCGATCGGAG[CCTCCTGGCCATGG
AAAACCCCA]GGCTTTGGGGAGCTGGATTCCCAAAGGGGAGGCTGCTCTGTGGGGTAGAGAGGGCCAGTGTGGT
GCGATCTTGTGGGAGGAGTTGAGATTGGATGCGTTGGGAAGTGTCAGTTTTGAGAGTCCTGAATGCCACGCCAGT
TTTTTGAACTCTTGTCAATAAGAAATGGAGAGAGTGGGGTCACAGGATGGAACACTGTAGAGGAGGAAGTGACT
GTCGGCAAGTGTGGAGAGAGGAGCCCCAGCTTCTGACCCAGGGCCCATAGAGAGTCCGTAAGGGGCCGTAGAA
GCCTGGTTAGAACATGTCTAGGAAGAGGCTGGAGATGGGCTTTCTT

sgRNA 1B

Off target site 1: NC_000008.11: 22539844-22540683 Homo sapiens chromosome 8, GRCh38.p7 Primary Assembly

AGGTCAGCCTCTTCAGCGATTCTAAAAGATTTCAAGAGCAGAGGCAGAAGTAGACTGGAATTTAGTTCAATTCATT
TCTGAGGTTGCCCTAAGGTAGGCAAGTTAAATTAACTTTGTTTCTATGAAAACCATTGGAACTGATAAACTTGTATT
ATACTTGTAATTGTTTTATTAGGTAAGATGGTTCAAAACGCGAATGCCTTAACTTGGTGTTATAAAAGTTGAATCGG
AAGGAAAATATGTAGAAGCTATTTTTAAGTTTTTAACTTATGTACAGCTCCTGGTTGTGCAAGATAGAAAGCTGTC
CTAAACGAAAAGATTAGCTCTTTTGTTACTTTTTTTTGTGGTCTATGTGGCCTTTGTGCGAAAAGCAACTACCCTGG
GTTTAACTTGTTCAATTATTCTAGTGTTGCCTTTGTTCTAGCATTGAGTATGTTTCTGGTGCATAAGACACTCTTTCTT
AAAGGTACTGGGGCCCAGTAAGTACTTGTTAGCTTTTTGTTAGGTATATGTCTACCATGATTATGCATATAGAGCTT
TTATGAAAATTACTTTCACAATTTCAGCTGCCCTTTGGGCTTTAAGGAAGCAGTAGCTGCAGCCAAGACTGATTCA
GTCAGACATTGCCCATGACTTTCACTAGACGTGTGCTCCATAGCCACCTTTCTGTGTGTTTCTCTAGGAGGTTGCTG
TGTGCTAAGAAACTTTTTTTAAGCCTGTTGCTTTGCTGCCTAATTGAGCTCTCTCCACCTGCTTCCTGTTTTTCTGTAG
[CCATCAGAGGGTTCTCGCTTCAG]CACAAGATCCGGAGTTTTGAAGAAGCGCGAGGTCTGGACCGAATTA

sgRNA 1B

Off target site 2: NC_000011.10: 112807711-112808876 Homo sapiens chromosome 11, GRCh38.p7 Primary Assembly

GAATGGTTCCATCAGGCCCAGATGGGGGCCTCAAAGAGCTGGTCAGCTGGACACCCTCTGACTCCTCCCTCCCCCA
TCCCTGTGCTTCCCTGGACCTGGGATCAAACCTATTTTCAGGGCAAACCTTGCAAGGGCTCCAAACTGGGAGAGAG
CGGGTGGGAGGGAGGTGCAAGAAGAGAGGGCACTTTGCTGTTTACAGCTAAGCCCTGGTGTCTGAAAGCCTCAA
ACTGAAGACATCAATAATTCATACATTCATATGCTGTAGAGTATTTCCCCTTTTGTCACATATCTGGCCAATTGATTT
CCCTTGGCCACAAACCTGCTGCTTTTAGAACATCATTAATAACAAGTCAACTGGTAAAATCTTCCCTGGGAGAGGG
GGCTTTGGTTGGGAGAGGTTCCTGACCTTGTTTCCCCCAAGAAAAGGGGCTGTGAGCAGCCCTGGCAGCAGAATG
TATGCACATGGGCTGGGAGCCTTCCTCCTCCTGTTTTAGTGGAAGAAGGGACTGGGGGCCAGAGAGATGAGATG
CCCACCCTATGGCCTCCCCAGG[CTATTGCTGGAACCCTCTGATAG]GGTTACAGGGAGAGAAGGAAAGAGAGAG
AGAGAGAGAACTTGGGAGCCTCAGAATAAGAATACAGGTTCCTAATGGAACCCTCTCTAGCAGCCTCCTCCTCCCC
TCCTCACCCCAGGTTCTGTCTCTGGGGGTTTCCGTGTCTGATCCTCAAGTACCACGGGATGAAACTAATCAGGGGC
TCTGGAATTACTCAAAGAGCTGGGAGCCCATGGTGCAACCTGGAGCTTCTACCAAAGCCTCCCTGCACCTCGTCAG
GGAAACCAGGAGGCCACCCAGCTGAGGCCCTCTTGGAGTGGCAAAATGGGGCAAGGGCATCTTTCCTGCTGGCT
CCATTCCCTGAGCCCCTTCCATGGCATCCGGAGCCCAGATTGTCTCCTCCTTCTGCTTCTGTGAGTCCCTGGACAGC
ACCTGGCTCGTAGGGAATGACCCTTCATGGATACTCACAAGAGACTTGTTGAAAGAGTAAATGAAAGCGGAAAGG
AACTGACAACTCATTCACAGTAAGTAAAGTGCTGCCACTTCCTCAGGACTCTGGGTTTACAGAGACTCAAAGGAAT
GAGTCTCTACTGGTGGGATGTCAGCAACTTGGGA

sgRNA 1B

Off target site 3: NC_000005.10: 72736962-72737995 Homo sapiens chromosome 5, GRCh38.p7 Primary Assembly

GGAAATCTAAGGGTACTGTAGGGTTAGGCCGGGGGTGGTGGCTCATGCCTGTAATCCTAGCACTTTGGGAGGCT
GAGGTGGGTGGATTGCCTAAGCTCAGGAGTTCGAGACCAGCTGGCCTAACATAATGAAACCCCATCTCTACTAAA
AATAAAAAAATAAAAATATTAGCCGGGTGTGCTGGGGTGCATCTGTAGTCCTAGCTACTCAGGAGACTGAGGCAT
GAGAATTGCTTGAACCCAGGAGGTAGAGGTTGCAGTGAGCTGAGATCGTGCCACTGTACTCCAGCCTGGGCGAC
AAAGCAAGATTCTGTCTCCCAAAAAAGAAAAAATAATAATTAATTAATTAAAAAGGTACTGTAGGGTTAAAACCAC
AGCCACGGAAGGGGAGAAGGAGAG[GAGAGGCAGGAACCCTCTGAAGG]CAGAGTCTGAAGGAGAACTGGGGA
AATTTGGAAGGGGAAAGATGCATCTTTGGGGGCAGTGGCTAGGATAAGAGAGTAAGAGAATGAAAGAAAGAAA
AAGAAGATTTTTACAATGCAGCTGTCCCTCAATATCTGCAAGCGATTGGTTCCAGGACCCCCAGGATACCAAAATT
TGCCAATGCTCAAGTCCCTGATAAAAATTGGCATAGAAGTTGCATATAACCTATGTGCATCCTCCTATATACTTCCA
GTCATCGCTAGATTACTTATAATACCTAATACAATGTAAGTGCTATGGAAATAGTTGTTACACGGTACTGTTTAGGG
AATAATGACACGAAAAAAAGTCTGTGAATGTTCAGTATGGAATTATCCTTTTTATTTTTTCTGAGTGGTTTTCCACCC
ACAGCTGGCTGAATCCAGGAACGTGGAACCCATGGATATGGAGGGCCAGCTGTATTGGCTGTGCCTTACAAATGA
AGTGCACCAGAGGCTACAATAAATATTCAATTGCTTTTCAATTACTATGGATAGTGAATTGAGTTCTCCCTTTTTCTT
AATGTGACACAATGCTGATCTGGGCCCACAAGTGGATGTGTCTTTCCGTGGGTTG

sgRNA 1B

Off target site 4: NC_000010.11: 90729730-90730789 Homo sapiens chromosome 10, GRCh38.p7 Primary Assembly

GCCTTAGCCAATTTGCTCATAAATCAAGGATTACCTGTATTTCAAATAATCACTGAATACCTCAAGAGGGAAGCTTC
TTTCTAAATAATTTCAGGTTTAAAAGAATCAGTTCAACCATTGTGGAAGTCAGTGTGGCGATTCCTCAGGGATCTA
GAACTAGAAATACCATTTGACCCAGCCATTCCATTACTGGGTATATACCCAAAGGACTATAAATCATGCTGCTATAA
AGACACATGCACACATATGTTTATTGCGGCACTATTCACAATAGCAAAGACTTGGAACCAACCCAAATGTCCAACA
ATGATAGACTGGATTAAGAAAATGTGGCACATATACACCATGGAATACTATGCAGCCATAAAAAAGGATGAGTTC
ATGTCCTTTGTAGGGACATGGATGAAATTGGAAATAGTCATTCTCAGTAAACTATCACAAGGACAAAAACCAAACA
CTGCATGTTCTCACTTATAGGTGGGAATTGAACAATGAGAACACATGGACACAGGAAGGGGAACATCACACTCTG
GGGACTGTTGTGGGGTGGGGGGAGGGGGGAGGGATAGCATTAGGAGATATACCTGATGCTAAATGACGAGTTA
ATGGGTGCAGCACACCAGCATGGCACATGTATACATATGTAACTAACCTGCACATTGTGCACATGTACCCTAAAAC
TTAAAGTATAATAATAATAAAATAAAAAAAAGAATCAGATCTTATTAAACCAATCAACATGACCCTAGGTTTTTAGT
AAAGTGATCAATTGCTTTTATTTTATATTTTTTATTCCTTTGTATCAAGTCACCTATAAATCTAGGCATGTAAAAATAT
GTAGTTCTCTTCATGTTGTCAGAAGTGTCCAGATGGTTTGACGCCTTACATGGAAAGAAAATCAGCAGTTAACTT[C
TAAAGAGGGAACCCTCTGGGAG]CAGATCAAAAATAAGAATGTTGTGTTAAGGGGAATATTTTTGGCTAAGGACT
AGATTGAGAATTAGAAGAGTAAAAGAAATTTAAAGGTGTTTTCCCCAAAATAAGCTGCAAAGGCCAACATCCT

sgRNA 1B

Off target site 5: NC_000023.11: 151153829-151155455 Homo sapiens chromosome X, GRCh38.p7 Primary Assembly

TCCGTTCCATCTTTTGCGGAGAGTGCCAGAGGCACACATCTCAGAGAGTCGTGACTGTGTCATTATGTTCTCGTCA
GCACAGATGATTCAGTCAATGTATAAGCAAGCACAGTTGCATTTTATACGACTTTCAAATCAGTTCATTTCCAAGTC
AGAAAAATCTTCCAGCAAGTCTGATCACCACACACACAGGGCAGCAACCAGTTCTAGGGGCTTCACCTGTTAAGAT
GATTTGGCTTGTTTGCAAGTCATTTTCAGAGAACAAGAGAGACAATCTTCCCACTACTCTATAATTAGAGTGCTGG
GGGCAGGCTGGGGGGAAGACAGCATGAACATGGAGCTCTCATTCTTATTCAGGAGCGTCTCTAATTCACACGGCC
CTGGGGACTGCTTCAGATGTAATATTCTCTCCACAAAGGATGGAAATCTTTTCTCTTAAAACGTCTCATCGCTGCAA
CTTTGCTGTAAGGCTTCATCGGATAAGTGTATAAAAAATCCAAAACTCACCTGTTTTCTTATTTCATCCAACTCTTTT
GTGTTTATATAATTAAGCACACCCTGCTTGATTGGTGTGTGTGTGGGATGTGTGTGTTTGTGTGTGTAAATCTTAAA
GGACTCAGAAGTTGGAGTGTTCGTGCGCTTATCACAGCTCCACGGACTCTATGTAATCATTTAGCTTCTTGAGGCTT
TTGCTGCCCTGTGATTTTCAGAAGTGCAGTGTCTTCATCACTCTTTGAACCCCCACAATGCCTGAGGCAGGGAGGC
AAACTACTGGACTTTTGGTAAATGACAATTTCATTCTGCAACAATTGGGTGGGAATATTCCAG[CTTCCAGAGGGTT
CCCACTTTCC]TGGTCTGTTAAATGGAACCAAGCCTACCTGCGTATCTTGTTGTGGGAGCTTCAGACTCTGCAGCAG
TTAAAGCCTTCATTTTTGGTCTTTACTTAACCACCCATAAGCTTCTTGTTCTGTTCCCTGCTCTTCTCCCAGTCCTTGC
CTTGCATCATTATTCTCTCTATACCTCAGACCTCTGCCCAACCTAAAGAGAAGCCTGTCTGGGGCCTTGCTTTGTTTT
TCCCTGTGTTTCTAACCCCCAGAATAGGGCCCGGAGCACGCAAGAGACTTGGGGCTGAATGAACGTAATTGACTT
GCTTGGATTCCCTGCACAGGTGACTGGGATCAGATCATTTTCCTTCTCAGAGACCTAGTTGTCTCACTTGTACAAGA
AGGGACTAACCTGGATGACTTCTGAAATCTTTTATTTTTGAGAAGAGGATTTCATAATTCTATGAATATTTTGTATTT
TTCTATATTTATTCACTTATCACTTTCTTCTTTCCATTCTTTCTAGCACAGAGGAAGGGGAACAGCGAGAAACAACCC
CCAAACAAGTGAACCCAAACACTAGTTCCTCTCTACCTTCCTAAAAATCATATTTTCTCTGTTGTACAATGGTTGTTT
TTTTCCTATCTTATCAAGAGTCATCCGTTCAGGTGATTTCAAGATGACTAATGGCCAAGTTGGTTGTTCATGCTGTA
CGCTCCTGCTGCTGGAACTGAGCACTTGAATGCTAATAATACTCTCCACGTCACCAATCTCATTTTTTTCTGACGTAC
TGACTGACCCCT

sgRNA 1B

Off target site 6: NC_000001.11:   6052625-6053681 Homo sapiens chromosome 1, GRCh38.p7 Primary Assembly

CTCTGCTAGCCACCGTTCTCTGGCTGGTGTAAATCCCTCTCAATTGCAAATTGACAGCTTTATCTTCTGGCCTCTCAG
GAACATGCCCTTGTGAGAGCCATTCAGCCAAAGAGGACCCTCCCCACCCAGTCCTTGTAGAGTTGCCAGTTAAATT
TGAAGTTCAGATAAACGCGTAACTTTTTAGCATAAATACGTTCCATGCAATATTTGGGTCATACTTATACTAAAAAG
TTCCTCATTGTTTATCTGAACTTCAAATTTAACTGGGCGTCCTGTGTTTTATCTGGCAACACTAAATATCTGGCTTCC
GTCTGAGACAGACCCTAGGGATCATCTGCGTGCTCTGTGCAGAGTGGAGTGCAGGCCAGGACCGCTCCTTTAACG
TTTTCTCCTGTGGACGGCGTGTTTGGTCCTTCAGCCCATTGTTTGAATATGTGGTTGTAATTTAGAAACAAAACACT
TCTCCTGTGAGTCCATTAGATGGCAGTGTTTTTCAGGCTTTGTATCCTAAATCCAGCTGTAAAGGAAGGACAAAGG
GTCCGCTAGTTTGGCCACAGCTGACCTCCTTGTTCTGCTGGAAATTTCTCAAGAAGGAGACGCTGGGCATTCATGA
GGGGCTGGTCATTGATTCCTTTTGTTTATTCAGTAAATGTTTATTGAGAGTCTGCTTGGCACCAGGCGCTGAGCCA
GATCCTGGAGCTGCTGCATGAACAAGAGCAACCAGGTCCCCAACCTCACGGCAGCCATGGCCTGGGGGTGTGGG
GGGCAGAGAAGCAGCGAGCTTGCACGGAGACAAGTTCCAACAGCGACCACTGGCCGAGGCAGCAAAGCAGGAC
AATGCAATGAAGAAAGACCAGGGAG[GCCGAGGGGGAACCCTCTGAGAG]AGGGATCATCTGAAGATGTGGAGA
ACATTCCAGCCCAAGAGGAGAGGGGTCTGGAATGGATGCCTCTCTCAGCTGGAGGAGCGAAGAGAAGGCCCAGG
CGGCTAGGCTGGAGGGAGGAAGGTGGAGGACACAAGGCGATGATGCAGGAGGGGCCAGACCCCATCCTCCAAA
C

sgRNA 1B

Off target site 7: NC_000023.11: 55279910-55280619 Homo sapiens chromosome X, GRCh38.p7 Primary Assembly

GGTGTCAGGAGAGCTTGTGGTCTAGGTTCTCCACAGAGGCAGGAAAACTTCAGTCACGTGGTGAATGCCCCACTC
AGTGCGCATGCTGCACAGATCTGTTACTGCAGATTTCTGTTGCTACGCATGTCTGTTACGCATGCGCCTTGTTGCCT
ACCTTCTGTCCATGCAGAGCTTTGTGAGAAGAGGTAGTATCTTCATTCTTTCCACCATCTTGATTCTTTCTCTCTGAC
TGAGGCTCAGCCGGTAGGTCCGCAGAGCGGTCTTCCTGGGAATTTAGTTGTGAGTGAATGTGAGGAGGAGCCAG
CGGGCTTTGGACAGGTCCTGCGGCACAGTCTTTGGCTTCTGAGGGAAAGGGGCCTCGCAGTCGTCGTCCGGCTCC
TCCCAGGTCGCAATGCTGCTATGGGCCTTGACGTAGTGGCTAGGCTGGAACTAGGGAGGAAAGTGGGCCGCAGA
GGGGAGGGATCACGTGAAGATGGGGCGAGTGCTGGAGGTGCTGTTAGAGGTATCTGAGTCC[CGAAAACTGGAA
CCCTCTGAGAG]AGGACAGTTTCCAGACTCCTCAGTAGGGACGCGGGAAGGGATGGTGAGGTCGGCAAGGAAGG
GGCCTGGGAACTAGGAACGCTGTGGGCTGGTGACTGCGGCCCTGAGGTCTGTAGAGTGCCTGGCAGAGGTGTCC
CGTGAGGAACATAAACTTCCCTCTGTGTCGCAACTTC

sgRNA 1B

Off target site 8: NC_000006.12:   61930823-61931624 Homo sapiens chromosome 6, GRCh38.p7 Primary Assembly

GATTGCCTGGAGGAAGCTAAGTATCAGGCTTTAGTAGAGCTCTGTTTCATTTAAATGTTAAACCTAATACCACATT
TTTTACTCAAAGAAAGATTATTCAAATTTAACAACTGCAATTTTTCTACTCAGGTGGATAGAAATATTCTGGTGACA
ATTTTTGTTTCAAATAAGCAACTCTTCTGCATATGTAATAAAATACCACCTAATTTTACATAATAATGTGAATCAACA
AAAAATTCAATCATATTACACATATACAATAAAATATTTTTATATTCATCCTTATAATGAAAAAAATACTTATATTTT
GTTAAAAATATAAGAAGTTTCATCCAAAATACTACTTACGCATCTTCACTGAAAATCACTATAAGAAAATAGGGCTT
GATTCCTTACATACTACATGAAAGGAGCTAAATAAAATTGATTTAGAGTATATGTTC[ATAAATCCGTAACCCTCTG
AAGG]TCATTTACTGAAACTTTCAGGTAACTAAACTTTTTATAAAGGTTGGAAGTTTTATAAAAGATGAAAGTATAG
TATTACCCAAAAGAAACTAAAGAAACCTGAAATGTTGGACTTAAAAACTATATAAAATATTAAAGATGTTAAGAAT
ATATACATACACACATATATATGTGCTAAAGATATATATATACATAATCCTTAACACTTGATAACATATTATCACTTA
TATAAAAATATACAATATTCAAAGAAGAATTTAAGTGGTTTTATATGCTCATTTATTAGTAATAAAGCTTTAAATAT
AATTTTATGCTTTTGAGAGAGTATTGCAAACT

sgRNA 1B

Off target site 9: NC_000010.11: 18772978-18774019 Homo sapiens chromosome 10, GRCh38.p7 Primary Assembly

AGCTGAGGAGGGTACTGAGGTGGGAAGACAATGGAGAGAGGCTTTCAGAAGGGAGGGAAGTGTTGCGTCTCCA
TCCTTGAGGCTGCAGATGATAGAGAATACCTGCCTTTCTGGGACAGTGGAGGGGCCGGAGTGATTGGGGTGGAA
CTTTCCCCTGCCATTCCAAGGGGCCTGAGGTCAGGGCATGGGCAGAACTCCAAGGTGCCACCTCCACAACAATAA
AGTGATTCCTTCCTTCAATCAAACAGACCCAATTACTGGGAGGAAAAAAACCAAGACATTCTAAACCAAGCCGTTT
GACAGGAGAAAGTTAAAATAGTCACCATGTCACTGCTGGA[GTCAAGCCTCAACCCTCTGACAG]GTTTTTAACAG
GTGAAATCTATTCCATTAGTAATTCCCTATAGGACACCATGACAGCTGCCTGAGCTCCCAGTTCCCATGTTTGCGCT
ATTATTTTCCTGAGGCCCCGTTGTTTTCCTTAGGCCCCAGATACAAACTTGGGATAATCAACAAACTAATTTTGTCCC
CTTTTTCGTGAACTCCCCAGCAGAGAATTACTCCACTTTTTTCAGCTCCAAGACACTGCCTTATCTGAAAGATGCTAA
TAGGATGGCATCTGCGTAGACAGGGATCATAAATATTCAGATCAATTCCATGTAGTACTTGTTATGTGCTTAGCTG
TGGCTCTGACAAAAGATGAGTAAAACATAGTTGTAATCCTCAGAGAAACAGGGTAATTGTTTGTGGGGGGAAATG
AGGAGATGCTAAAAATTAAGCATGAAGGACTCTTATGTTTACAGGAGAATTCTACACCAAAACGCTAAAATTCAG
GGAGATACATAGCAAAGGACCCAAATGGGCCCACTAGAGAAGGAATTTTAGTATATATGTGTGGGGTAAGTCAG
ATCGGTGTGGGTTTGTGGTAATACAGGAAGGCTTTATGAATGAATTGGGACTTGGTCTTTAGGAATGAATAGGAG
TTGGGTCGACAGAGTGGTTGGTGAGAGGACATTCCAGAAGGAGAAGCAGGACAGCGAGTGT

sgRNA 1B

Off target site 10: NC_000008.11: 51422814-51423616 Homo sapiens chromosome 8, GRCh38.p7 Primary Assembly

CCTTCAATGCCTCTCCGTGTAGAGAACACACCAGGCAATCTTAACAGCCTGTGTCCACTTGTAAATAACCTCAGCTG
AGTTTCAGTACAGTCTACTCAAGTCTGCCTACAAATGGACTCTCCCATGTCCCTTGTGAACTGAATAATCAAAAAAA
GAAAAAATATATTTTTTATAAGACTACAGTAAACAGTATTTTAAACAGTCATTATATATAATCAA[CCTGCAGAGGG
TTCCAGCTATAT]AACCAGATTGGTGATAGAATGTAAAAAGCTATGTCAATTATGTTGGCCAATTATAAATGCCTGG
AAACACTGAGATATTTGTTCTAGTGGTTGTAAAGTTTACTTGGGAGTTTGATGGATTGATTTCTAATTTTAAACTGG
GATTAAATATACTAGGCTAGAGTATGTAGTACATGTTGAATTCCATATTATGCTTCCACCTCCGGCTCCACATTTTG
GCTATAAATACATCTTGCATTCTATTATTTTTGGTTTGTAAGAAAACATGATATATATTTGCAAATCTTTGCTATTTAT
GTTCTTTTTTTAATTTGTTATTTTTCACTTTATATCCTTTAAGATTGTTTATATTTAAATTTTTTTATTTTAAAAAGAATA
AAACAGCTTTGACTATAAACACACTTGACCAGAAAGAAAGTATAAGAGAGCATGATTAGTGAGATCAGTCAAATA
GGATTGGGGTAGAAGCTGTTCAAAGACAGTTATTTGGATGTTGTAAATGGAGCTATAGACACCTACCCGTGACTG
TAAGAAACATGTTGGTCACAGCAAGGCCAAAAG
